# *Bradyrhizobium ontarionense* sp. nov., a novel bacterial symbiont isolated from *Aeschynomene indica* (Indian jointvetch), harbours photosynthesis, nitrogen fixation and nitrous oxide (N_2_O) reductase genes

**DOI:** 10.1007/s10482-024-01940-6

**Published:** 2024-04-22

**Authors:** Eden S. P. Bromfield, Sylvie Cloutier

**Affiliations:** Agriculture and Agri-Food Canada, 960 Carling Ave., Ottawa, K1A 0C6 Canada

**Keywords:** *Aeschynomene indica* (Indian jointvetch), *Bradyrhizobium ontarionense* sp. nov., Nitrous oxide reductase gene, Photosynthetic gene cluster, Symbiotic nitrogen fixation

## Abstract

A novel bacterial symbiont, strain A19^T^, was previously isolated from a root-nodule of *Aeschynomene indica* and assigned to a new lineage in the photosynthetic clade of the genus *Bradyrhizobium.* Here data are presented for the detailed genomic and taxonomic analyses of novel strain A19^T^. Emphasis is placed on the analysis of genes of practical or ecological significance (photosynthesis, nitrous oxide reductase and nitrogen fixation genes). Phylogenomic analysis of whole genome sequences as well as 50 single-copy core gene sequences placed A19^T^ in a highly supported lineage distinct from described *Bradyrhizobium* species with *B. oligotrophicum* as the closest relative. The digital DNA-DNA hybridization and average nucleotide identity values for A19^T^ in pair-wise comparisons with close relatives were far lower than the respective threshold values of 70% and ~ 96% for definition of species boundaries. The complete genome of A19^T^ consists of a single 8.44 Mbp chromosome and contains a photosynthesis gene cluster, nitrogen-fixation genes and genes encoding a complete denitrifying enzyme system including nitrous oxide reductase implicated in the reduction of N_2_O, a potent greenhouse gas, to inert dinitrogen. Nodulation and type III secretion system genes, needed for nodulation by most rhizobia, were not detected. Data for multiple phenotypic tests complemented the sequence-based analyses. Strain A19^T^ elicits nitrogen-fixing nodules on stems and roots of *A. indica* plants but not on soybeans or *Macroptilium atropurpureum*. Based on the data presented, a new species named *Bradyrhizobium ontarionense* sp. nov. is proposed with strain A19^T^ (= LMG 32638^T^ = HAMBI 3761^T^) as the type strain.

**Supplementary Information:**

The online version contains supplementary material available at 10.1007/s10482-024-01940-6.

## Introduction

Bacteria belonging to the genus *Bradyrhizobium* represent one of the most abundant taxa in soils globally and are considered a priority group for research on furthering the understanding of the contribution of soil microbes to ecosystem functioning (Delgado-Baquerizo et al. 2018). Moreover, the genus *Bradyrhizobium* includes economically important species that fix nitrogen in symbiotic association with agricultural plants such as soybean (*Glycine max*) and peanut (*Arachis hypogaea*) (Lindström and Mousavi 2019), species that are capable of photosynthesis (Giraud et al. 2007), and, species that possess genes for nitrous oxide (N_2_O) reductase and are able to reduce N_2_O, a potent greenhouse gas, to dinitrogen (N_2_) (Minamisawa 2023).

Bacterial species possessing photosynthesis genes are distributed across the genus *Bradyrhizobium* (Avontuur et al. 2023). These include apparently free-living (non-symbiotic) species such as *B. betae* (Rivas et al. 2004; Cloutier and Bromfield 2019), *B. amphicarpaeae* (Bromfield et al. 2019) and *B. cosmicum* (Wasai-Hara et al. 2020b). Others are represented by the species, *B. oligotrophicum* (Okubo et al. 2013; Ramirez-Bahena et al. 2013), ‘*B. aeschynomenes’* (Sun et al. 2022), and, *B. denitrificans* (van Berkum et al. 2006) that are symbionts of tropical plant species of the genus *Aeschynomene*, and based on core gene analyses, are placed exclusively in the so called “photosynthetic clade” (Avontuur et al. 2023) of the genus *Bradyrhizobium*. These bacterial species lack the Type III Secretion System (T3SS) and nodulation (*nod*) genes needed for symbiosis by most rhizobia and yet are still capable of eliciting root- and stem-nodules on *Aeschynomene* species in a nodulation (nod) factor -T3SS independent manner (Giraud et al. 2007; Camuel et al. 2023).

In previous work (unpublished) we grew plants of *Aeschynomene indica* (Indian jointvetch) in pots in the glasshouse to produce seed for our research*.* After several weeks of plant growth in the glasshouse, a few sporadic nodules were observed on plant roots that were apparently due to “volunteer” bacteria in the rooting medium. Analysis of *recA* house-keeping (core) gene sequences placed bacteria isolated from these root-nodules in several novel lineages in the genus *Bradyrhizobium.*

The objective of the current work was the detailed genomic, phylogenetic and phenotypic description of one of these lineages represented by strain A19^T^. The novel strain, placed in the “photosynthetic clade” of the genus *Bradyrhizobium* possesses photosynthesis, nitrogen fixation and nitrous oxide (N_2_O) reductase genes and is capable of eliciting nitrogen-fixing nodules on the stems and roots of *A. indica* plants. Based on the data presented, a new species is proposed with the name *Bradyrhizobium ontarionense* sp. nov.

## Materials and methods

### Bacterial strains

Bacterial strain A19^T^ was isolated from a nodule that formed on the root of an *Aeschynomene indica* plant raised from surface sterilized seed and grown in a pot in a glasshouse at Agriculture and Agri-Food Canada, Ottawa for six weeks (16 h, 25 − 28 °C (day); 8 h, 16 − 18 °C, (night)). The rooting medium consisted of a mixture of Canadian peat moss and locally sourced (Ottawa, Ontario) black-earth that had been sterilized by steaming for 8 h and then stored in bulk without aseptic precautions until use.

Bacteria employed in this work are listed in Table S1 or in the Tables and Figures of the main text and Appendix. Bacterial strains were grown on modified yeast extract-mannitol (YEM) agar medium having the following composition (g/l^−1^): yeast-extract (Thermo Scientific™ Oxoid™), 1.5; mannitol, 1.0; NaCl, 0.1; K_2_HPO_4_, 0.5; MgSO_4_· 7H_2_O, 0.2; Bacteriological agar (Thermo Scientific™ Oxoid™), 18.0. Bacterial cultures were maintained in 20% w/v glycerol at − 80 °C.

### Genomic DNA sequencing and phylogenetic analysis

Genomic DNAs were extracted and purified from bacterial cells grown for 7 days at 28 °C on YEM agar medium as detailed by Bromfield et al. (2023).

Sequencing of the genome of strain A19^T^ was done at the Genome Quebec Innovation Centre, Canada, employing Pacific Biosciences (PacBio) Sequel Single-Molecule Real-Time (SMRT) technology (Ardui et al. 2018). Flye software (version 2.9) (Kolmogorov et al. 2019) was used for genome sequence assembly.

Analysis of the core gene sequences (*atpD*, *glnII*, *gyrB*, *recA*, *rpoB* and 16S rRNA) of strain A19^T^ together with type strains of *Bradyrhizobium* species and widely studied members of the “photosynthetic clade” (*Bradyrhizobium* spp. strains BTAi1, ORS278 and ORS285 (Giraud et al 2007; Renier et al 2011)) were done using sequences retrieved from whole genome sequences (where available). Analysis of photosynthesis reaction center (*pufLM*) genes, nitrogen fixation (*nifHDK*) genes and the nitrous oxide reductase (*nosZ*) gene were done using full-length gene sequences retrieved from genome sequences. Alignment of nucleotide sequences was performed using MUSCLE (Edgar 2004). Sequence accession numbers are listed in Table S1.

Further analysis was carried out using 50 single-copy core gene sequences encoding ribosome protein subunits (*rps*) of novel strain A19^T^ and 77 *Bradyrhizobium* reference strains (Jolley et al. 2012). Aligned and concatenated sequences of *rps* genes of A19^T^ and reference strains were obtained from genome sequences using the Genome Comparator tool implemented in the domain genome database of the BIGSdb software platform (Jolley and Maiden 2010). To avoid confounding the phylogenetic analysis, the sequences *rpsU*, *rpmH* and *rpmJ* were excluded because they were either incomplete or paralogous in several *Bradyrhizobium* reference strains.

The ModelTest-NG tool (Darriba et al. 2020) in the web-based CIPRES Science Gateway version 3.3 (Miller et al. 2010) was used to select best fit substitution models. Phylogenetic analyses using MrBayes (software version 3.2.1) were done employing default priors as described previously (Yu et al. 2014). Maximum-likelihood analyses were performed using 1,000 non-parametric bootstrap replications (Guindon et al. 2010). Bayesian trees are only presented in this work as trees reconstructed from Bayesian and Maximum-likelihood methods exhibited similar topologies (results not shown).

A whole genome sequence based phylogenetic tree was reconstructed using the online Type Strain Genome Server (TYGS) (Lefort et al. 2015; Meier-Kolthoff and Göker 2019).

### Genomic analyses

The overall genome relatedness indices of digital DNA–DNA hybridization (dDDH) and average nucleotide identity (ANI) are routinely employed in bacterial taxonomic studies to facilitate species circumscription (Chun et al. 2018; Meier-Kolthoff and Göker 2019). Algorithms implemented in the TYGS were used to calculate dDDH values and associated confidence intervals (Holland et al. 2002; Meier-Kolthoff et al. 2013; Meier-Kolthoff and Göker 2019). The accepted dDDH threshold of 70% was used to define species boundaries (Meier-Kolthoff and Göker 2019). ANI values were calculated utilizing FastANI (Jain et al. 2018) performed in the K base web server (Arkin et al. 2018). The ANI threshold ~ 96% was used for delineation of bacterial species boundaries (Richter and Rosselló-Móra 2009; Lee et al. 2016; Ciufo et al. 2018).

Genome sequence comparisons were facilitated by utilizing the software, Geneious Prime 2023.0.4 (https://www.geneious.com) and GenomeMatcher (Ohtsubo et al. 2008).

### Phenotypic characterisation

The Gram-stain reactivity of bacterial cells was assessed using the protocol outlined by Buck (1982).

Analysis of fatty acids, was done using bacteria grown at 28 °C on YEM agar medium for 7 days. Fatty acids were extracted as detailed by Sasser (1990). Identification of fatty acids was performed using the Sherlock Microbial Identification System (MIDI) version 6.0 and RTSBA6 database.

Tests of chemical sensitivity and carbon source utilization were done using BIOLOG GEN III MicroPlates (Biolog™, United States) as detailed in the manufacturer’s instructions.

Cells of strain A19^T^ were examined using a scanning electron microscope (model, Hitachi SU7000 FESEM) and a transmission electron microscope (model, H-7000; Hitachi). For microscopic examination of cells, strain A19^T^ was grown for 96 h in YEM broth at 28 °C as outlined previously (Bromfield et al. 2023).

Tests of acid or alkali production by A19^T^ and reference strains grown for 21 days on YEM agar medium at 28 °C were performed as detailed by Bromfield et al. (2010).

Plant tests were carried out with modified Leonard jars (Vincent 1970) (two plants per jar with three replicate jars for each inoculation treatment) using nitrogen-free nutrient solution as detailed by Bromfield et al. (2010). Seeds of *A. indica* were vernalized by immersion in liquid nitrogen (− 196 °C) for 20 s and surface sterilized by serial immersion in 70% ethanol (30 s), 10.5% sodium hypochlorite solution (90 s) followed by multiple washes in water over two hours. The seeds were left in sterile water overnight at 4 °C to facilitate imbibition and then transferred to water agar plates (Bacteriological agar (Thermo Scientific™ Oxoid™) 15 g/l) at 25 °C to germinate. For tests of *A. indica* stem nodulation, cell suspensions of *Bradyrhizobium* test strains (ca. 10^9^ cells/ ml in sterile water) were applied to stems with cotton-wool swabs. The inoculated portions of stems were kept moist for 48 h using wrappings of moistened tissue paper covered with plastic cling film.

Nodulation and symbiotic nitrogen fixation were assessed by visual comparison of shoots and roots of plants inoculated with A19^T^ (as test strain) relative to negative control plants (uninoculated) and positive control plants inoculated with an effective strain: *Bradyrhizobium* sp. BTAi1 (for *A. indica* plants) and *B. diazoefficiens* USDA110 (for *Glycine max* (soybeans) and *Macroptilium atropurpureum* ‘siratro’). Symbiotic nitrogen fixation was considered to be ‘effective’ based on the size and colour of shoots (*i.e.,* large, green and healthy) and size and number of root-nodules possessing pink pigmented interiors (characteristic of leghaemoglobin, a phytoglobin necessary for symbiotic nitrogen fixation).

## Results and discussion

### Genomic and phylogenetic characterisation

A complete genome sequence of novel strain A19^T^ was generated in this work; genome coverage was 634-fold with 60,448 polymerase reads and 91,671 bp average read length. The genome of strain A19^T^ consists of a single chromosome of size 8,435,845 bp and has a DNA G+C content of 64.9 mol% (Table 1).

**Table 1:** Genome characteristics of *Bradyrhizobium ontarionense* sp. nov. A19^T^ and reference strains

Characteristic	Strain										
	*B. ontarionense* sp. nov A19^T^	*B. oligotrophicum* S58^T^	*‘B. aeschynomenes’* 83002^T^	*Bradyrhizobium* sp. BTAi1	*Bradyrhizobium* sp. ORS 278	*Bradyrhizobium* sp. ORS 285	*‘B. guangzhouense’* CCBAU 51670^T^	*B. amphicarpaeae* 39S1MB^T^	*B. betae* PL7HG1^T^	*B. cosmicum* 58S1^T^	*B. japonicum* USDA 6^T^
Genome assembly (no. contigs)	Complete (1)	Complete (1)	Draft (58)	Complete (2)	Complete (1)	Complete (1)	Complete (2)	Complete (1)	Complete (2)	Complete (1)	Complete (1)
Genome size (bp)	8435845	8264165	7522254	8493513	7456587	7797098	8138177	7044517	7419402	7304136	9207384
Plasmid no. (size, bp)	0	0	na	1 (228826)	0	0	1 (979291)*	0	1 (269307)	0	0
Genes (total)	7456	7276	6808	7700	6624	6937	7683	6604	7167	6985	8652
CDSs (total)	7390	7212	6739	7638	6672	6877	7629	6547	6780	6757	8438
G+C content %	64.9	65.1	65	64.8	65.5	65.2	63.4	64.7	64.4	64.0	63.7
No. of rRNA operons (5S, 23S, 16S)	2	2	na	2	2	2	1	2	1	1	2
Photosynthetic gene cluster	Yes	Yes	Yes	Yes	Yes	Yes	Yes	Yes	Yes	Yes	No
Nodulation *(nodABC)* genes	No	No	No	No	No	Yes	Yes	No	No	No	Yes
Nitrogen fixation* (nifHDK*) genes	Yes	Yes	Yes	Yes	Yes	Yes	Yes	Yes	No	Yes	Yes
Type III Secretion System (T3SS) genes	No	No	No	No	No	Yes	Yes	No	No	No	Yes
Nitrous oxide reductase (*nosRZDFYL*) genes	Yes	Yes	Yes	Yes	No	Yes	No	No	Yes	Yes	No
tRNAs	56	54	51	52	52	50	47	50	47	48	56

^*^Symbiosis plasmid

na, data not available

The analysis of 16S rRNA gene sequences has traditionally been used as a taxonomic tool in species descriptions. However, this gene is highly conserved and different bacterial species may possess identical 16S rRNA sequences (Richter and Rosselló-Móra 2009; de Lajudie et al. 2019). Nevertheless, the analysis of 16S rRNA gene sequences is considered to be useful for verifying the genus level identity of bacteria (Young et al. 2023).

The phylogenetic tree of 16S rRNA gene sequences (Fig. S1) of strain A19^T^ and 86 *Bradyrhizobium* species (type strains) confirms placement of A19^T^ in the genus *Bradyrhizobium*. The tree also shows that strain A19^T^ is placed in a novel lineage with *B. oligotrophicum* as the most closely related species.

Multiple Locus Sequence Analysis (MLSA) of single copy, protein encoding gene sequences is a widely used tool for species differentiation (Jolley et al. 2012; de Lajudie et al. 2019).

The Bayesian tree of concatenated core gene sequences (*atpD*-*glnII*-*gyrB*-*recA*-*rpoB*; alignment length, 2679 positions) of strain A19^T^ and reference strains (Fig. S2), corroborates the placement of A19^T^ in a new *Bradyrhizobium* lineage with *B. oligotrophicum* S58^T^ as the closest relative. Fig. S2 also shows that strain A19^T^ is placed in the “photosynthetic clade” (represented by *B. oligotrophicum*) containing photosynthetic symbionts of the tropical legume *A. indica*. It should be noted that *Bradyrhizobium* sp. BTAi1 shares a lineage with the type strain of *B. denitrificans* and therefore represents a potential member of this species. Moreover, strains ORS278 and ORS285 are placed in distinct lineages and represent potential genospecies.

The taxonomic status of strain A19^T^ was further investigated by MLSA of 50 single-copy core gene sequences consisting of concatenated full-length bacterial ribosome protein subunit (*rps*) gene sequences (Jolley et al. 2012) as well as a phylogenomic analysis (based on whole genome sequences) implemented in the TYGS (Meier-Kolthoff and Göker 2019). A genome sequence of reference strain, *B. denitrificans* IFAM 1005^T^, is not available in public databases. As our phylogenetic analysis of five concatenated core gene sequences (Fig. S2) showed that the widely studied strain, *Bradyrhizobium* sp. BTAi1, is a potential member of the species, *B. denitrificans*, we used BTAi1 as a proxy for IFAM 1005^T^ in subsequent phylogenetic and genomic analyses.

**Fig. 1:**
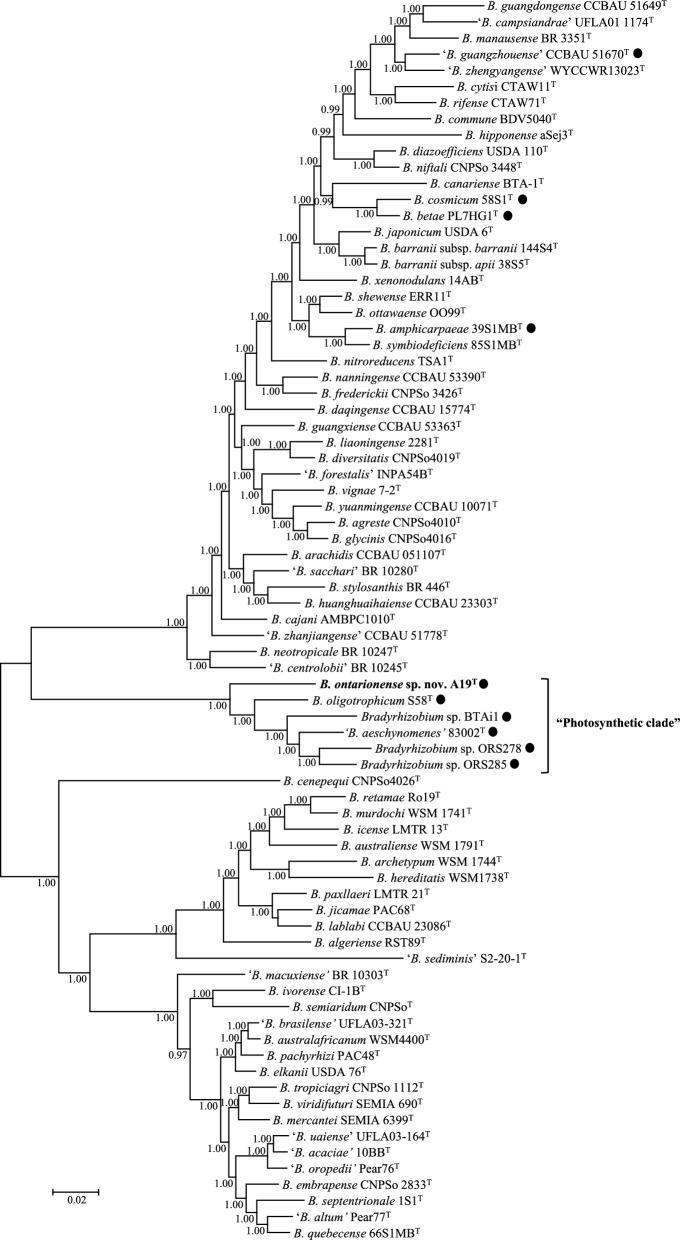
Bayesian tree (GTR+G+I substitution model) inferred from 50 full-length concatenated ribosome protein subunit (*rps*) gene sequences of *Bradyrhizobium ontarionense* sp. nov. strain A19^T^ and reference taxa. Alignment length, 22,500 bp. Black dots designate species possessing photosynthesis genes. Posterior probabilities ≥ 0.90 are indicated. Bar represents expected substitutions per site

The phylogenetic tree of 50 core (*rps*) gene sequences (Fig. 1) and the TYGS tree based on whole genome sequences (Fig. S3) support the finding that strain A19^T^ is placed in a highly supported novel lineage within the “photosynthetic clade” represented by *B. oligotrophicum*; *B. oligotrophicum* is also the closest relative.

Table 2 shows dDDH and ANI values for pair-wise comparison of the genome sequence of strain A19^T^ with the genome sequences of five reference strains that are placed in the *Bradyrhizobium* “photosynthetic clade”. The largest values in these comparisons (33.4% (dDDH) and 88.8% (ANI)) are far lower than the threshold values (70% and ~ 96%, respectively) used for the definition of species boundaries. Based on these results, strain A19^T^ represents a new species of *Bradyrhizobium,* with *B. oligotrophicum* as the most closely related species.

**Table 2:** ANI and dDDH values for pair-wise comparisons of the genome sequence of *Bradyrhizobium ontarionense* sp. nov A19^T^ (Accession no. CP088156) with members of the “photosynthetic clade”

Reference strain (sequence accession no.)	Fast ANI^*^	dDDH % [C.I.]^†^
	A19^T^	A19^T^
*B. oligotrophicum* S58^T^ (AP012603)	88.8	33.4 [31.0–35.9]
‘*B. aeschynomenes*’ 83002^T^ (JABFDM01)	87.5	30.7 [28.3–33.2]
*Bradyrhizobium* sp. BTAi1 (CP00494-CP000495)	86.8	30.2 [27.8–32.7]
*Bradyrhizobium* sp. ORS 285 (LT859959)	87.1	29.8 [27.4–32.3]
*Bradyrhizobium* sp. ORS 278 (CU234118)	86.9	29.4 [27.0–31.9]

^*^Values represent averages of reciprocal comparisons

^†^dDDH values and confidence intervals [C.I.] based on Genome BLAST Distance Phylogeny (GBDP) formula 4 implemented in the Type Strain Genome Server (TYGS) (Meier-Kolthoff and Göker 2019); formula 4 is independent of genome length and is robust against use of incomplete draft genomes

Genome sequence analyses revealed that strain A19^T^ possesses a photosynthesis gene cluster (PGC) of size about 49 kb (co-ordinates 3,498,729–3,547,553 bp). The PGC contains key photosynthesis genes encoding bacteriochlorophyll (*bchIDOCXYZGPFNBHLM and acsF*), reaction centre L, M and H subunits (*pufLM and puhA*), light-harvesting protein alpha and beta subunits (*pufBA*), carotenoid (*crtIBCDEF*), bacteriophytochrome (*bphP*) and photosynthesis repressor (*ppsR1* and *ppsR2*) proteins.

**Fig. 2:**
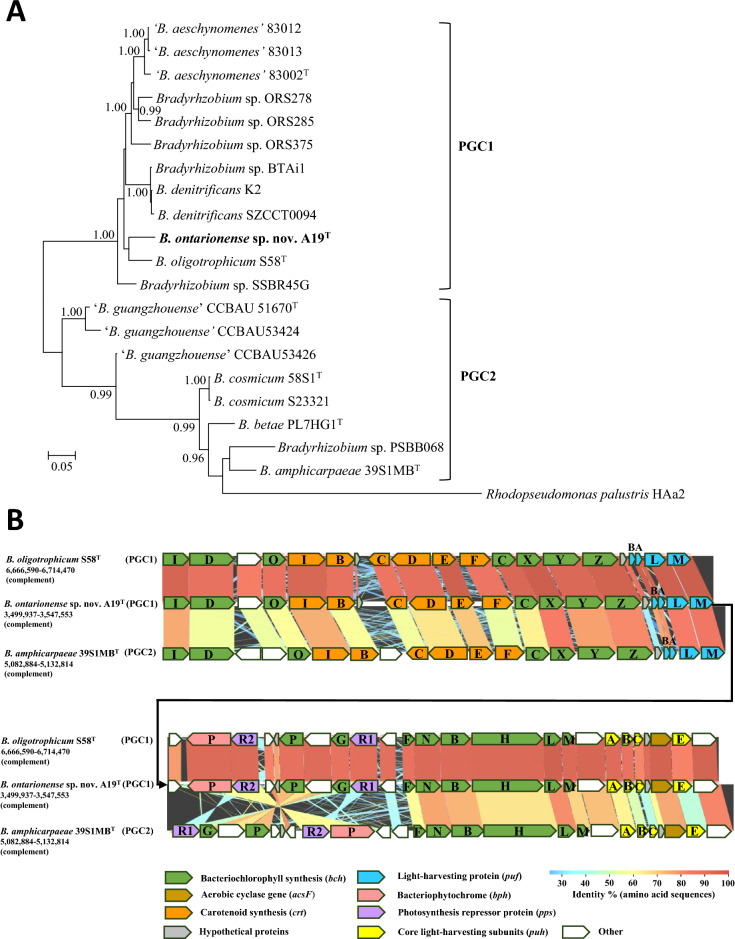
**A** Bayesian phylogenetic tree (GTR+G+I substitution model) of concatenated photosynthetic reaction centre, *pufLM*, genes (1739 bp) for *Bradyrhizobium ontarionense* sp. nov*.* strain A19^T^ and reference taxa showing division of *Bradyrhizobium* strains into two clades corresponding to photosynthetic gene cluster type 1 (PGC1) and type 2 (PGC2) as defined by Avontuur et al. (2023). **B** Comparative arrangement of the photosynthesis gene cluster of *Bradyrhizobium ontarionense* sp. nov*.* strain A19^T^ (PGC1) relative to *Bradyrhizobium*. *oligotrophicum* (PGC1) and *Bradyrhizobium amphicarpaeae* (PGC2). Color coding represents % identity based on amino acid sequences of PGC genes calculated by GenomeMatcher software (Ohtsubo et al. 2008)

A Bayesian phylogenetic tree of concatenated photosynthetic reaction centre, *pufLM*, genes (Fig. 2A) shows that strain A19^T^ is placed in a cluster together with other symbionts of *A. indica*; the closest relative of A19^T^ is *B. oligotrophicum* S58^T^. The *pufLM* gene tree also shows that non-symbiotic species (*B. cosmicum*, *B. amphicarpeae* and *B. betae)* are placed in a separate cluster (clade) from the *A. indica* symbionts (*B. denitrificans*, *B. oligotrophicum*, ‘*B. aeschynomenes’ and Bradyrhizobium* sp*.* BTAi1 and novel strain A19^T^). It is notable that the phylogenetic division of symbiotic and nonsymbiotic bradyrhizobia on the basis of *puf* gene analysis corresponds to the two types of photosynthetic gene clusters (PGC1 and PGC2, respectively) defined by Avontuur et al. (2023). The organization of genes in the PGC of strain A19^T^ is similar to close relative *B. oligotrophicum* possessing a type 1 PGC but differs from non-symbiotic bacteria such as *B. amphicarpaea* carrying type 2 PGCs (Fig. 2B).

Further analyses show that novel strain A19^T^ lacks key nodulation (*nodABC*) and Type III Secretion System (T3SS) genes (Table 1) indicating that its symbiotic association with *A. indica* plants is initiated in a nod-factor and T3SS independent manner similar to the well characterised photosynthetic strains, *Bradyrhizobium* spp*.* BTAi1 and ORS278 (Giraud et al. 2007; Camuel et al. 2023).

In contrast to the absence of *nod* genes, the following key nitrogen fixation genes were found in the genome of strain A19^T^: *nifDKEN, nifH, nifA* and *fixABCX* (co-ordinates 7,793,281–7,842,331 bp). The phylogenetic tree of concatenated full length *nifHDK* gene sequences (Fig. S4) shows that strain A19^T^ occupies a lineage that is well separated from other *Bradyrhizobium* species; the type strains of ‘*B. aeschynomenes’* and *B. oligotrophicum* are closest relatives.

It should be noted that to date novel strain A19^T^ represents only the fourth species to be placed in the “photosynthetic” clade (based on core gene sequence analysis—see Fig. 1) and as such represents a useful resource to further investigate the evolution of photosynthesis and symbiosis traits in the genus *Bradyrhizobium*.

Agricultural soils are a major source of N_2_O, a highly potent greenhouse gas and accelerant of ozone layer depletion (Montzka et al. 2011; Tian et al. 2020). The majority of N_2_O released from soils originates as a byproduct from the respiratory activity of nitrifying and denitrifying microorganisms (Thomson et al. 2012). While N_2_O can be generated by multiple mechanisms, the only known biological sink for N_2_O is the reduction of N_2_O to dinitrogen by the enzyme N_2_O reductase (encoded by the *nosZ* gene) found in some denitrifying bacteria (Torres et al. 2016; Minamisawa 2023). The *nosZ* gene has been detected infrequently in the rhizobia and has been found only in strains of the symbiotic species *B. diazoefficiens* (Sameshima-Saito et al. 2006; Itakura et al. 2013; Akiyama et al. 2016), *B. ottawaense* (Wasai-Hara et al. 2020a, b), *Ensifer meliloti* (Bueno, et al. 2015) and *Rhizobium leguminosarum* (Hénault et al. 2022). Recently *nosZ* gene containing strains of the nitrogen-fixing soybean symbiont, *B. ottawaense*, were found to be highly efficient with regard to the reduction of N_2_O to inert dinitrogen gas and the use of these strains as inoculants was suggested as a strategy for mitigating N_2_O emissions from agricultural soils (Wasai-Hara et al. 2023). In the current study we detected key genes encoding enzymes required for the complete denitrification of nitrate or nitrite to nitrogen gas in the genome sequence of strain A19^T^ as follows: *napEDABC* (nitrate reductase); *nirK* (nitrite reductase); *norCBQDE* (nitric oxide reductase) and *nosRZDFYLX* (nitrous oxide reductase). Based on these findings, novel strain A19^T^ represents a new resource for furthering studies on the reduction of N_2_O to inert gaseous nitrogen by members of the genus *Bradyrhizobium.*

We carried out further analyses to assess the frequency of occurrence of the *nosZ* gene (encoding N_2_O reductase) in the genus *Bradyrhizobium* by screening the genome sequences of type strains of named species. The results (Table S2) show that contrary to earlier reports, a substantial minority (i.e., 21 of 73 *Bradyrhizobium* species type strains) possess the *nosZ* gene. It is noteworthy that, with the exception of *Bradyrhizobium* sp. ORS 278, symbionts of the aquatic legume *A. indica* (*B. oligotrophicum* S58^T^,*‘B. aeschynomenes’* 83002^T^, novel strain A19^T^ (Table 1 and Table S2) and *Bradyrhizobium* spp. strains BTAi1 and ORS 285 (Table 1)) possess the *nosZ* gene, suggesting that reduction of N_2_O to nitrogen (where nitrate rather than oxygen is used as a terminal electron acceptor during respiration) might be an adaptation to oxygen limitation in environments subject to periodic waterlogging.

A Bayesian phylogenetic tree of the *nosZ* gene of strain A19^T^ and reference strains of the genus *Bradyrhizobium* is presented in Fig. S5. The placement of strain A19^T^ in a distinct lineage (closest neighbours, *B. xenonodulans, B. lablabi* and *B. zhenyangense*) is incongruent with its placement in trees based on core genes (Fig. 1) and whole genome sequences (Fig. S3) (closest neighbours *B. oligotrophicum* and* ‘B. aeschynomenes’*), suggesting that the *nosZ* gene was acquired by horizontal gene transfer from external sources.

*B. denitrificans* IFAM 1005^T^, a member of the “photosynthetic clade” and symbiont of the tropical legume, *A. indica*, was originally isolated from surface lake water in Germany (Hirsch and Müller 1985). Although novel strain A19^T^ was also isolated in a temperate region (from a root-nodule of a tropical *A. indica* plant), we can only speculate as to its origin. The *A. indica* plants used in the present work had been raised from surface sterilized seeds and planted in rooting medium that was sterilized making it unlikely that A19^T^ had been initially introduced on seed or in the rooting medium. However, as plants were maintained in a greenhouse without aseptic precautions it is possible that A19^T^ was later accidentally introduced into the rooting medium by watering, fertilization or by aerial contamination from other sources such as soybeans (*G. max*) or corn (*Zea mays*) that had been grown in nearby facilities.

### Phenotypic analyses

**Fig. 3:**
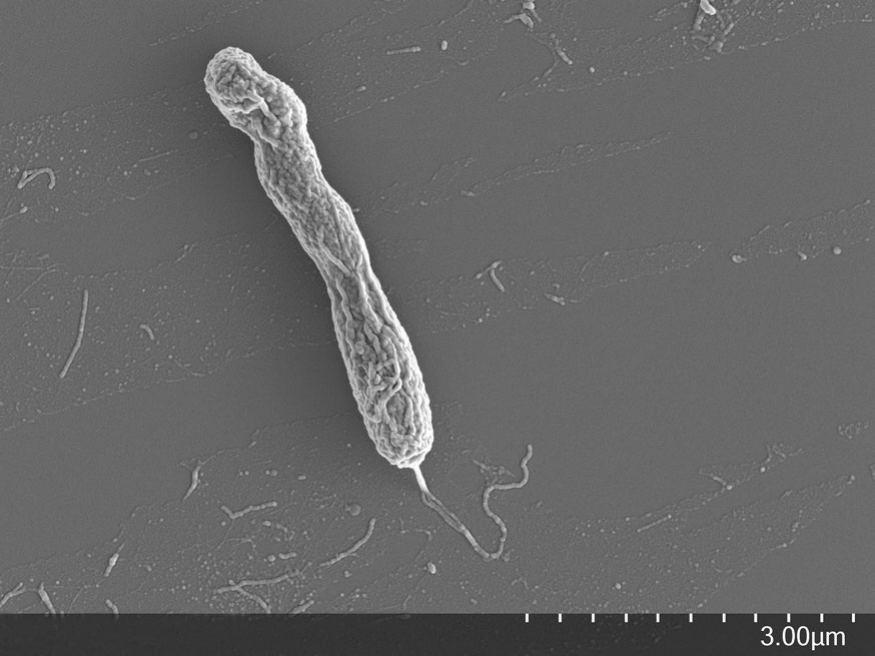
Scanning electron microscope image showing morphological features of a cell of *Bradyrhizobium ontarionense sp.* nov A19^T^. Scale bar (μm) is indicated

Colonies of strain A19^T^ are circular, cream coloured, raised and with diameters ~ 0.5 mm after growth on YEM agar medium for 7 days at 28 °C. Cells of strain A19^T^ are Gram-stain-negative, rod shaped, and based on examination by electron microscopy, possess at least one flagellum (Fig. 3 and Fig. S6). Growth on YEM agar medium at 28 °C is accompanied by an alkaline reaction (Table S3), which is characteristic of the genus *Bradyrhizobium*. Strain A19^T^, like close relative *B. oligotrophicum* S58^T^, shows growth at pH 5, but does not grow at pH 10, at 10 °C, or, in the presence of 0.5% NaCl, after 7 days incubation on YEM agar medium. However, strain A19^T^ differed from *B. oligotrophicum* S58^T^, in that it did not grow at 37 °C (Table S3).

Strain A19^T^ produced pink-pigmented colonies on modified HM agar medium (Okubo et al. 2013) after 7 days at 28 °C under natural light (14 h light, 10 h dark), typical of photosynthetic reference strains, *B. oligotrophicum* S58^T^ and *Bradyrhizobium* sp. BTAi1.

Results for fatty acid profiles of strain A19^T^ and four reference strains are presented in Table S4. Fatty acids C16:0, 18:1ω7c 11-methyl and C18:1 ω6c/C18:1 ω7c (summed feature 8), were detected in A19^T^ and all four reference strains. The dominance of fatty acids C16:0 and C18:1 ω6c/C18:1 ω7c (summed feature 8) in strain A19^T^ is typical of the genus *Bradyrhizobium* (Tighe et al. 2000).

Table S5 shows the results for assays of carbon source utilization and chemical sensitivity utilizing Biolog™ phenotype microarrays. The data show that strain A19^T^ can be readily differentiated from photosynthetic symbionts of *A. indica* (*B. oligotrophicum* S58^T^, *B. denitrificans* IFAM 1005^T^ and ‘*B. aeschynomenes’* 83002^T^), as well as from the (non-photosynthetic) genus type strain (*B. japonicum* USDA6^T^) based on multiple tests.

**Fig. 4:**
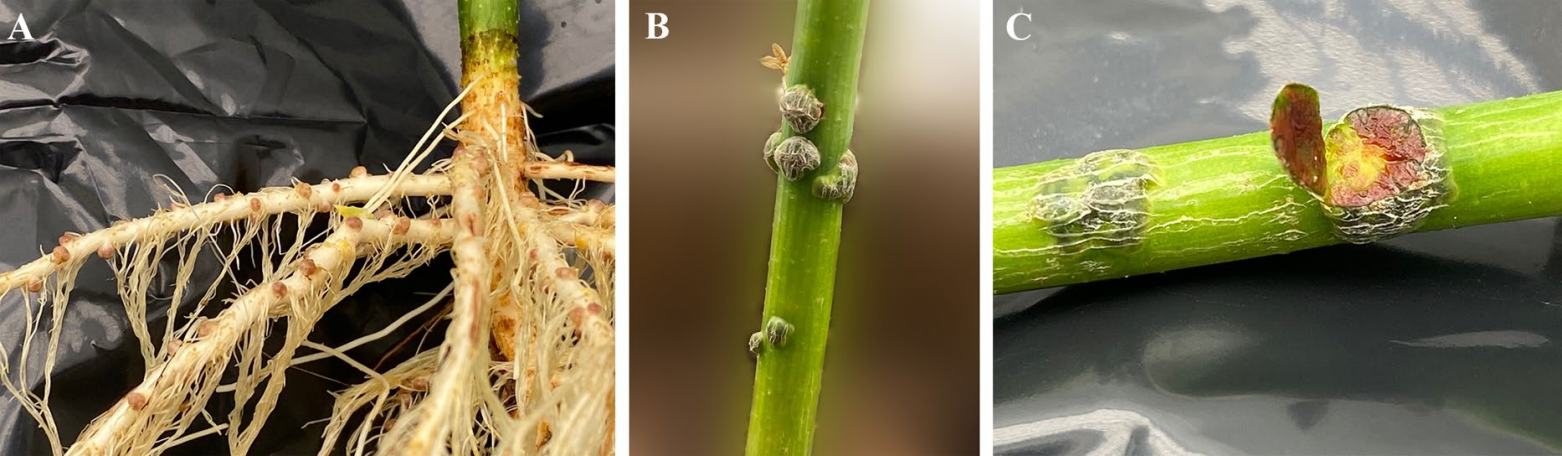
Effective nitrogen-fixing root (**A**) and stem (**B** and **C**) nodules on *Aeschynomene indica* plants inoculated with *Bradyrhizobium ontarionense* sp. nov. A19^T^. Panel (**C**) shows the cut surface of an effective stem nodule exhibiting red colored pigment characteristic of leghaemoglobin that is required for nitrogen fixation

Plant tests showed that strain A19^T^ was able to elicit efficient nitrogen fixing nodules on the stems and roots of *A. indica* plants (Fig. 4) but did not form nodules on ‘Glengarry’ soybeans or *Macroptilium atropurpureum* ‘siratro’.

### Description of *Bradyrhizobium ontarionense *sp. nov.

*Bradyrhizobium ontarionense *(on.ta.ri.o.nen′se. N.L. neut. adj. *ontarionense*, of or belonging to the province of Ontario). Bacterial cells are aerobic, non-spore-forming rods, Gram-stain-negative and possess one or more flagella. Colonies on YEM agar medium are cream colored, raised and circular with diameters ~ 0.5 mm after growth for 7 days at 28 °C. Produces an alkaline reaction on YEM agar medium. Grows at pH 5 but not at pH 10 (optimum ~ pH 7.0). Does not grow at 10 °C or 37 °C (optimal at ~ 28 °C) or in the presence of 0.5% (w/v) NaCl. Produces pink-pigmented colonies on modified HM agar medium after 7 days of light–dark cycles at 28 °C. Dominant fatty acids are C16:0 and C18:1 ω6c/C18:1 ω7c (summed feature 8). The type strain is able to utilize 17 carbon sources including α-d-Glucose, d-Galactose, d-Sorbitol, d-Mannitol, d-Arabitol, d-Gluconic Acid, d-Malic Acid, l-Malic Acid, Tween 40, Propionic Acid, Acetic Acid and Formic Acid. The type strain does not utilize 53 carbon sources including d-Fructose, l-Fucose, myo-Inositol, d-Glucose- 6-PO4, d-Fructose- 6-PO4, d-Aspartic Acid, Gelatin, Pectin, d-Galacturonic Acid, l-Galactonic Acid Lactone, Mucic Acid, l-Lactic Acid, Citric Acid, and γ-Amino-Butryric Acid. The type strain is resistant to 1% Sodium Lactate, Troleandomycin, Rifamycin SV, Minocycline, Lincomycin, Tetrazolium Violet, Tetrazolium Blue, Nalidixic Acid and Aztreonam. Susceptible to Fusidic Acid, Niaproof 4, d-Serine, Guanidine HCl, Vancomycin, Potassium Tellurite, Lithium Chloride, Sodium Butyrate and Sodium Bromate.

Elicits efficient nitrogen fixing root- and stem-nodules on plants of the aquatic legume *Aeschynomene indica.* Does not elicit nodules *G. max* (soybeans) or *Macroptilium atropurpureum*.

The type strain, A19^T^ (= LMG 32638^T^ = HAMBI 3761^T^) was isolated from a root-nodule of an *Aeschynomene indica* plant grown in a greenhouse. The size of the genome is 8.44 Mbp and the DNA G+C content is 64.9 mol%. The type strain does not possess nodulation or type III secretion system genes but contains photosynthesis genes, nitrogen-fixation genes and genes encoding a complete denitrifying enzyme system including nitrous oxide reductase.

## Supplementary Information

Below is the link to the electronic supplementary material.

Supplementary file1 (PDF 1046 KB)

## Data Availability

All data required for this study are included in this paper and Supplementary Information. The whole genome shotgun project for *Bradyrhizobium ontarionense* sp. nov. strain A19^T^ was deposited at DDBJ/ENA/ GenBank as accession number CP088156. Raw PacBio data was deposited in the NCBI Sequence Read Archive as BioProject accession number PRJNA783021. A culture of *Bradyrhizobium ontarionense* sp. nov. strain A19^T^ was deposited in the Culture Collection of Bacteria (BCCM/LMG), University of Ghent, Belgium as LMG 32638^T^ and in the Microbial Culture Collection (HAMBI), University of Helsinki, Finland as HAMBI 3761^T^.

## Abbreviations

ANI: Average nucleotide identity
dDDH: Digital DNA DNA hybridization
GBDP: Genome blast distance phylogeny
MLSA: Multiple locus sequence analysis
TYGS: Type strain genome server
YEM: Yeast-extract mannitol medium
