# Supplementary material for: Bradyrhizobium ontarionense sp. nov., a novel bacterial symbiont isolated from Aeschynomene indica (Indian jointvetch), harbours photosynthesis, nitrogen fixation and nitrous oxide (N2O) reductase genes
Source: Antonie Van Leeuwenhoek. 2024 Apr 22;117(1):69. doi: 10.1007/s10482-024-01940-6 (PMC11035471; doi:10.1007/s10482-024-01940-6)
Supplement: Supplementary file 1 — Supplementary file1 (PDF 1046 KB) [file 10482_2024_1940_MOESM1_ESM.pdf]

Supplementary Materials

Title: *Bradyrhizobium ontarionense* sp. nov. isolated from *Aeschynomene indica* (Indian jointvetch) harbours photosynthesis, nitrogen fixation and nitrous oxide (N_2O) reductase genes.

Authors: Eden S. P. Bromfield* and Sylvie Cloutier

Affiliation: Agriculture and Agri-Food Canada, 960 Carling Ave., Ottawa, K1A 0C6, Canada.

***Correspondence**: Eden S. P. Bromfield; Email: eden.bromfield@agr.gc.ca

Table S1 GenBank nucleotide accession numbers for *Bradyrhizobium ontarionense* sp. nov. A19^{T} and reference taxa.

Strain	*atpD*	*glnII*	*gyrB*	*recA*	*rpoB*	*16S rRNA*	*nifHDK*	*nosZ*	*pufML*
***B. ontarionense* sp. nov. A19^{T}**	CP088156	CP088156	CP088156	CP088156	CP088156	CP088156	CP088156	CP088156	CP088156
'B. acaciae' 10BB	JACMYK01	JACMYK01	JACMYK01	JACMYK01	JACMYK01	JACMYK01	JACMYK01	NA	NA
'B. aeschynomenes' 83002^{T}	JABFDM01	JABFDM01	JABFDM01	JABFDM01	JABFDM01	JABFDM01	JABFDM01	JABFDM01	JABFDM01
'B. aeschynomenes' 83012	NA	NA	NA	NA	NA	NA	NA	NA	JABFDN01
'B. aeschynomenes' 83013	NA	NA	NA	NA	NA	NA	NA	NA	JABFDO01
B. agreste CNPSo 4010^{T}	JACCHP01	JACCHP01	JACCHP01	JACCHP01	JACCHP01	JACCHP01	JACCHP01	NA	NA
B. algeriense RST89^{T}	PYCM01	PYCM01	PYCM01	PYCM01	PYCM01	PYCM01	PYCM01	NA	NA
'B. altum' Pear77	JACMYP01	JACMYP01	JACMYP01	JACMYP01	JACMYP01	JACMYP01	JACMYP01	NA	NA
B. americanum CMVU44^{T}	-	-	NA	-	NA	KU991833	NA	NA	NA
B. amphicarpaeae 39S1MB^{T}	CP029426	CP029426	CP029426	CP029426	CP029426	CP029426	CP029426	NA	CP029426
B. arachidis CCBAU 051107^{T}	FPBQ01	FPBQ01	FPBQ01	FPBQ01	FPBQ01	FPBQ01	FPBQ01	NA	NA
B. archetypum WSM1744^{T}	JAAVLW01	JAAVLW01	JAAVLW01	JAAVLW01	JAAVLW01	JAAVLW01	JAAVLW01	NA	NA
B. australafricanum WSM4400^{T}	JAGKJL01	JAGKJL01	JAGKJL01	JAGKJL01	JAGKJL01	JAGKJL01	JAGKJL01	NA	NA
B. australiense WSM1791^{T}	JAAVLX01	JAAVLX01	JAAVLX01	JAAVLX01	JAAVLX01	JAAVLX01	JAAVLX01	NA	NA
B. barranii 144S4^{T} subsp. *barranii*	CP086136	CP086136	CP086136	CP086136	CP086136	CP086136	CP086136	NA	NA
B. barranii 38S5^{T} subsp. *apii*	CP096251	CP096251	CP096251	CP096251	CP096251	CP096251	CP096251	NA	NA
B. betae PL7HG1^{T}	CP044543	CP044543	CP044543	CP044543	CP044543	CP044543	NA	CP044543	CP044543
'B. brasilense' UFLA 03-321^{T}	MPVQ01	MPVQ01	MPVQ01	MPVQ01	MPVQ01	MPVQ01	MPVQ01	NA	NA
B. cajani AMBPC1010^{T}	WQNE01	WQNE01	WQNE01	WQNE01	WQNE01	WQNE01	WQNE01	NA	NA
'B. campsiandrae' UFLA 01-1174^{T}	JAANIH01	JAANIH01	JAANIH01	JAANIH01	JAANIH01	JAANIH01	JAANIH01	NA	NA
B. canariense BTA-1^{T}	VSST01	VSST01	VSST01	VSST01	VSST01	VSST01	VSST01	NA	NA
B. cenepequi CNPSo4026^{T}	JAGKJI01	JAGKJI01	JAGKJI01	JAGKJI01	JAGKJI01	JAGKJI01	JAGKJI01	JAGKJI01	NA
'B. centrolobii' BR 10245^{T}	LUUB01	LUUB01	LUUB01	LUUB01	LUUB01	LUUB01	LUUB01	LUUB01	NA
'B. centrosematis' A9^{T}	-	-	NA	-	NA	KC247115	NA	NA	NA
B. cosmicum 58S1^{T}	CP041656	CP041656	CP041656	CP041656	CP041656	CP041656	CP041656	CP041656	CP041656
B. cosmicum S23321	NA	NA	NA	NA	NA	NA	NA	NA	AP012279
B. commune BDV5040^{T}	CP061379	CP061379	CP061379	CP061379	CP061379	CP061379	NA	NA	NA
B. cytisi CTAW11^{T}	VSSR01	VSSR01	VSSR01	VSSR01	VSSR01	VSSR01	VSSR01	NA	NA
B. daqingense CCBAU 15774^{T}	CP088014	CP088014	CP088014	CP088014	CP088014	CP088014	CP088014	NA	NA
B. denitrificans IFAM 1005^{T}	FM253153	HM047121	FM253239	EU665419	FM253282	NR_041827	NA	NA	NA
B. denitrificans K2	NA	NA	NA	NA	NA	NA	NA	NA	JAMKCE01
B. denitrificans SZCCT0094	NA	NA	NA	NA	NA	NA	NA	NA	JAFCLK01
B. diazoefficiens USDA 110^{T}	BA000040	BA000040	BA000040	BA000040	BA000040	BA000040	BA000040	BA000040	NA
B. diversitatis CNPSo 4019^{T}	JACEGD01	JACEGD01	JACEGD01	JACEGD01	JACEGD01	JACEGD01	JACEGD01	NA	NA

Strain	*atpD*	*glnII*	*gyrB*	*recA*	*rpoB*	*16S rRNA*	*nifHDK*	*nosZ*	*pufML*
B. elkanii USDA 76ᵀ	ARAG01	ARAG01	ARAG01	ARAG01	ARAG01	ARAG01	ARAG01	NA	NA
B. embrapense CNPSo 2833ᵀ	LFIP02	LFIP02	LFIP02	LFIP02	LFIP02	LFIP02	LFIP02	NA	NA
B. erythrophlei CCBAU 53325ᵀ	NA	-	-	-	-	KF1144645	NA	NA	NA
B. ferriligni CCBAU 51502ᵀ	NA	-	-	-	-	KX683400	NA	NA	NA
'B. forestalis' INPA54Bᵀ	PGVG01	PGVG01	PGVG01	PGVG01	PGVG01	PGVG01	PGVG01	PGVG01	NA
B. frederickii CNPSo 3426ᵀ	SPQS01	SPQS01	SPQS01	SPQS01	SPQS01	SPQS01	SPQS01	SPQS01	NA
B. ganzhouense RITF806ᵀ	-	-	NA	-	NA	JQ796661	NA	NA	NA
B. glycinis CNPSo 4016ᵀ	JACCHQ01	JACCHQ01	JACCHQ01	JACCHQ01	JACCHQ01	JACCHQ01	JACCHQ01	NA	NA
B. guangdongense CCBAU 51649ᵀ	CP030051	CP030051	CP030051	CP030051	CP030051	CP030051	CP030052	CP030052	NA
B. guangxiense CCBAU 53363ᵀ	CP022219	CP022219	CP022219	CP022219	CP022219	CP022219	CP022219 + CP02220	CP022219	NA
'B. guangzhouense' CCBAU 51670ᵀ	CP030053	CP030053	CP030053	CP030053	CP030053	CP030053	CP030054	NA	CP030053
'B. guangzhouense' CCBAU 53424	NA	NA	NA	NA	NA	NA	NA	NA	CP030053
'B. guangzhouense' CCBAU 53426	NA	NA	NA	NA	NA	NA	NA	NA	RDQZ01
B. hereditatis WSM1738ᵀ	JAGKJK01	JAGKJK01	JAGKJK01	JAGKJK01	JAGKJK01	JAGKJK01	JAGKJK01	NA	NA
B. hipponense aSej3ᵀ	VSTH01	VSTH01	VSTH01	VSTH01	VSTH01	VSTH01	VSTH01	NA	NA
B. huanghuaihaiense CCBAU 23303ᵀ	VLLA01	VLLA01	VLLA01	VLLA01	VLLA01	VLLA01	VLLA01	NA	NA
B. icense LMTR 13ᵀ	CP016428	CP016428	CP016428	CP016428	CP016428	CP016428	CP016428	NA	NA
B. ingae BR 10250ᵀ	-	-	-	-	-	KF927043	NA	NA	NA
B. iriomotense EK05ᵀ	AB300994	AB300995	HQ873308	AB300996	HQ587646	AB300992	NA	NA	NA
B. ivorense CI-1Bᵀ	CAADFC02	CAADFC02	CAADFC02	CAADFC02	CAADFC02	CAADFC02	CAADFC02	NA	NA
B. japonicum USDA 6ᵀ	AP012206	AP012206	AP012206	AP012206	AP012206	AP012206	AP012206	NA	NA
B. jicamae PAC68ᵀ	LLXZ01	LLXZ01	LLXZ01	LLXZ01	LLXZ01	LLXZ01	LLXZ01	NA	NA
B. kavangense 14-3ᵀ	KY753592	KM378446	KX661397	KM378399	KM378311	KP899562	NA	NA	NA
B. lablabi CCBAU 23086ᵀ	LLYB01	LLYB01	LLYB01	LLYB01	LLYB01	LLYB01	LLYB01	LLYB01	NA
B. liaoningense 2281ᵀ	FM253137	AY386775	FM253223	AY591564	FM253266	AJ250813	NA	NA	NA
B. lupini USDA 3051ᵀ	-	-	-	-	NA	KM114861	NA	NA	NA
'B. macuxiense' BR 10303ᵀ	LNCU01	LNCU01	LNCU01	LNCU01	LNCU01	LNCU01	LNCU01	NA	NA
B. manausense BR 3351ᵀ	LJYG01	LJYG01	LJYG01	LJYG01	LJYG01	LJYG01	LJYG01	NA	NA
B. mercantei SEMIA 6399ᵀ	MKFI01	MKFI01	MKFI01	MKFI01	MKFI01	MKFI01	MKFI01	NA	NA
B. murdochi WSM 1741ᵀ	AXAU01	AXAU01	AXAU01	AXAU01	AXAU01	AXAU01	AXAU01	NA	NA
B. namibiense 5-10ᵀ	KX661387	KM378440	KX661393	KM378377	KM378306	KX661401	NA	NA	NA
B. nanningense CCBAU 53390ᵀ	LBJC01	LBJC01	LBJC01	LBJC01	LBJC01	LBJC01	LBJC01	LBJC01	NA
B. neotropical BR 10247ᵀ	LSEF01	LSEF01	LSEF01	LSEF01	LSEF01	LSEF01	LSEF01	LSEF01	NA
B. niftali CNPSo 3448ᵀ	SPQT01	SPQT01	SPQT01	SPQT01	SPQT01	SPQT01	SPQT01	NA	NA
B. nitroreducens TSA1ᵀ	LFJC01	LFJC01	LFJC01	LFJC01	LFJC01	LFJC01	LFJC01	LFJC01	NA

Strain	*atpD*	*glnII*	*gyrB*	*recA*	*rpoB*	*16S rRNA*	*nifHDK*	*nosZ*	*pufML*
B. oligotrophicum S58^T	AP012603	AP012603	AP012603	AP012603	AP012603	AP012603	AP012603	AP012603	AP012603
'B. oropedii' Pear76^T	JACMYO01	JACMYO01	JACMYO01	JACMYO01	JACMYO01	JACMYO01	JACMYO01	NA	NA
B. ottawaense OO99^T	CP029425	CP029425	CP029425	CP029425	CP029425	CP029425	CP029425	CP029425	NA
B. pachyrhizi PAC48^T	LFIQ01	LFIQ01	LFIQ01	LFIQ01	LFIQ01	LFIQ01	LFIQ01	NA	NA
B. paxllaeri LMTR 21^T	CP042968	CP042968	CP042968	CP042968	CP042968	CP042968	CP042968	NA	NA
B. quebecense 66S1MB^T	CP088022	CP088022	CP088022	CP088022	CP088022	CP088022	CP088022	NA	NA
B. retamae Ro19^T	LLYA01	LLYA01	LLYA01	LLYA01	LLYA01	LLYA01	LLYA01	NA	NA
B. rifense CTAW71^T	VSSS01	VSSS01	VSSS01	VSSS01	VSSS01	VSSS01	VSSS01	NA	NA
B. ripae WR4^T	NA	-	-	-	-	MF593082	NA	NA	NA
'B. sacchari' BR 10280^T	LWIG01	LWIG01	LWIG01	LWIG01	LWIG01	LWIG01	LWIG01	NA	NA
'B. sediminis' S2-20-1^T	CP076134	CP076134	CP076134	CP076134	CP076134	CP076134	CP076134	NA	NA
B. semiaridum CNPSo^T	JAGKJJ01	JAGKJJ01	JAGKJJ01	JAGKJJ01	JAGKJJ01	JAGKJJ01	NA	NA	NA
B. septentrionalis 1S1^T	CP088285	CP088285	CP088285	CP088285	CP088285	CP088285	CP088285	NA	NA
B. shewense ERR11^T	FMAI01	FMAI01	FMAI01	FMAI01	FMAI01	FMAI01	FMAI01	FMAI01	NA
B. stylosanthis BR 446^T	LVEM01	LVEM01	LVEM01	LVEM01	LVEM01	LVEM01	LVEM01	LVEM01	NA
B. subterraneum 58 2-1^T	-	-	-	-	-	KP308152	NA	NA	NA
B. symbiodeficiens 85S1MB^T	CP029427	CP029427	CP029427	CP029427	CP029427	CP029427	NA	NA	NA
B. tropiciagri CNPSo 1112^T	LFLZ01	LFLZ01	LFLZ01	LFLZ01	LFLZ01	LFLZ01	LFLZ01	NA	NA
'B. uaiense' UFLA03-164^T	VKHP01	VKHP01	VKHP01	VKHP01	VKHP01	VKHP01	VKHP01	NA	NA
'B. valentinum' LmjM3^T	LLXX01	LLXX01	LLXX01	LLXX01	LLXX01	LLXX01	LLXX01	NA	NA
B. vignae 7-2^T	RDQF01	RDQF01	RDQF01	RDQF01	RDQF01	RDQF01	RDQF01	NA	NA
B. viridifuturi SEMIA 690^T	LGTB01	LGTB01	LGTB01	LGTB01	LGTB01	LGTB01	LGTB01	NA	NA
'B. xenonodulans' 14AB	CP089391	CP089391	CP089391	CP089391	CP089391	CP089391	CP089391	CP089391	NA
B. yuanmingense CCBAU 10071^T	FMAE01	FMAE01	FMAE01	FMAE01	FMAE01	FMAE01	FMAE01	NA	NA
'B. zhanjiangense' CCBAU 51778^T	CP022221	CP022221	CP022221	CP022221	CP022221	CP022221	CP022221	NA	NA
'B. zhengyangense' WYCCWR13023^T	JAKLTY01	JAKLTY01	JAKLTY01	JAKLTY01	JAKLTY01	JAKLTY01	JAKLTY01	JAKLTY01	NA
Bradyrhizobium sp. BTAi1	CP000494	CP000494	CP000494	CP000494	CP000494	CP000494	CP000494	CP000494	CP000494
Bradyrhizobium sp. ORS278	CU234118	CU234118	CU234118	CU234118	CU234118	CU234118	CU234118	NA	CU234118
Bradyrhizobium sp. ORS285	LT859959	LT859959	LT859959	LT859959	LT859959	LT859959	LT859959	LT859959	LT859959
Bradyrhizobium sp. ORS375	NA	NA	NA	NA	NA	NA	NA	NA	CAFI01
Bradyrhizobium sp. PSBB068	NA	NA	NA	NA	NA	NA	NA	NA	CP069300
Bradyrhizobium sp. SSBR45G	NA	NA	NA	NA	NA	NA	NA	NA	BSCT01
Rhodopseudomonas palustris HAa2	NA	NA	NA	NA	NA	NA	NA	NA	CP000250

NA, Not applicable, or, sequence not available in public databases.

- , Sequence not used in phylogenetic analyses either because sequence too short or not available in database

Table S2 Occurrence of the *nosZ* gene in genome sequences of species type strains of the genus *Bradyrhizobium*.

Species and type strain	Genome sequence accession no.	Symbiosis with legume host
***nosZ* gene present:**		
B. ontarionense sp. nov. A19^T	CP088156	Yes
'B. aeschynomenes' 83002^T	JABFDM	Yes
B. betae PL7HG1^T	CP044543	No
B. cenepequi CNPSo 4026^T	JAGKJI	Yes
'B. centrolobii' BR10245^T	LUUB	Yes
B. cosmicum 58S1^T	CP041656	No
B. diazoefficiens USDA 110^T	CP011360	Yes
'B. forestalis' INPA54B^T	PGVG	Yes
*B. fredericki*i CNPSo 3426^T	SPQS	Yes
B. guangdongense CCBAU 51649^T	CP030051-CP030052 (plasmid)	Yes
B. guangxiense CCBAU 53363^T	CP022219-CP022220 (plasmid)	Yes
B. lablabi CCBAU 23086^T	LLYB	Yes
B. nanningense CCBAU 53390^T	LBJC	Yes
B. neotropicale BR10247^T	LSEF	Yes
B. nitroreducens TSA1^T	LFJC	No
B. oligotrophicum S58^T	AP012603	Yes
B. ottawaense OO99^T	CP029425	Yes
B. shewense ERR11^T	FMAI	Yes
B. stylosanthis BR446^T	LVEM	Yes
'B. xenonodulans' 14AB	CP089391	Yes
B. zhengyangense WYCCWR 13023^T	JAKLTY	Yes
***nos*Z gene absent:**		
'B. acaciae' 10BB^T	JACMYK	Yes
B. agreste CNPSo 4010^T	JACCHP	Yes
B. algeriense RST89^T	PYCM	Yes
'B. altum' Pear77^T	JACMYP	Yes
B. amphicarpaeae 39S1MB^T	CP029426	No
B. arachidis CCBAU 051107^T	FPBQ	Yes
B. archetypum WSM 1744^T	JAAVLW	Yes
B. australiense WSM 1791^T	JAAVLX	Yes
B. australafricanum WSM 4400^T	JAGKJL	Yes
B. barranii 144S4^T	CP086136-CP086139	Yes
'B. brasilense' UFLA03-321^T	MPVQ00000000	Yes
B.cajani AMBPC1010^T	WQNE	Yes
'B. campsiandrae' UFLA 01-1174^T	JAANIH	No
B. canariense BTA-1^T	VSST	Yes
B. commune BDV5040^T	CP061379	Yes
B. cytisi CTAW11^T	VSSR	Yes
B. daqingense CCBAU 15774^T	CP0882014	Yes
B. diversitatis CNPSo 4019^T	JACEGD	Yes
B. elkanii USDA 76^T	ARAG	Yes
B. embrapense CNPSo 2833^T	LFIP	Yes
B. glycinis CNPSo 4016^T	JACCHQ	Yes
'B. guangzhouense' CCBAU 51670^T	CP030053-CP030054	Yes
B. hereditatis WSM 1738^T	JAGKJK	Yes
B. hipponense aSej3^T	VSTH	Yes
B. huanghuaihaiense CCBAU 23303^T	VLLA	Yes
B. icense HAMBI 3584^T	CP016428	Yes
B. ivorense CI-1B^T	CAADFC	Yes
B. japonicum USDA6^T	AP012206	Yes
B. jicamae PAC68^T	NZ_LLXZ	Yes
'B. macuxiense' BR10303^T	LNCU	Yes
B. manausense BR3351^T	LJYG	Yes
B. mercantei SEMIA 6399^T	MKFI	No
B. murdochi WSM 1741^T	AXAU	Yes
B. niftali CNPSo 3448^T	SPQT	Yes
'B. oropedii' Pear76^T	JACMYO	Yes
B. pachyrhizi PAC48^T	LFIQ	Yes
B. paxllaeri LMTR21^T	CP042968	Yes
B. quebecense 66S1MB^T	CP088022-CP088025	Yes
B. retamae Ro19^T	LLYA	Yes
B. rifense CTAW71^T	VSSS	Yes
'B. sacchari' BR10280^T	LWIG	Yes
'B. sediminis' S2-20-1^T	CP076134	No
B. septentrionale 1S1^T	CP088284-CP088287	Yes
B. semiaridum WSM 1704^T	JAGKJJ	No
B. symbiodeficiens 85S1MB^T	CP029427	No
B. tropiciagri CNPSo 1112^T	LFLZ	Yes
'B. uaiense' UFLA03-164^T	VKHP	Yes
'B. valentinum' LmjM3^T	LLXX	Yes
B. vignae 7-2^T	RDQF	Yes
B. viridifuturi SEMIA 690^T	LGTB	Yes
B. yuanmingense CCBAU 10071^T	FMAE	Yes
'B. zhanjiangense' CCBAU 51778^T	CP022221	Yes

Table S3 Growth characteristics of **1**. *B. ontarionense* sp. nov. A19^T, **2.** *B. oligotrophicum* S58^T*,* **3**. *'B. aeschynomenes'* 83002^T, **4.** *B. denitrificans* IFAM 1005^T, and, **5**. *B. japonicum* USDA6^T.

**Characteristic**	**1**	**2**	**3**	**4**	**5**
Growth on YEM agar medium*					
10 °C	-	-	±	-	±
37 °C	-	+	+	+	-
pH 5	+	+	+	+	+
pH 10	-	-	+	±	+
0.5% NaCl	-	-	±	-	±
1% NaCl	-	-	±	-	±
2% NaCl	-	-	-	-	-
Acid production †	7.5	ND	ND	ND	ND

* Positive, +; weak, ± ; negative, − ; not determined, ND. Values are based on three replicates.

† Acid production on YEM agar medium determined as described by Bromfield et al., (2010).

Table S4 Fatty acid profiles of *Bradyrhizobium* strains: **1**. *B. ontarionense* sp. nov., A19^T, **2.** *B. oligotrophicum* S58^T, **3.** *'B. aeschynomenes'* 83002^T, **4.** *B. denitrificans* IFAM 1005^T, and, **5**. *B. japonicum* USDA6^T.

Fatty Acid	1	2	3	4	5†
12:0	0.7	0.5		0.4	
14:0	0.3	0.3	0.3	0.1	
16:0	16.9	16.0	15.2	15.6	13.1
16:1 ω5c					3.6
16:1 ω11c	0.3			0.1	
17:1 ω8c		0.5	0.5		
17:1 ω6c		0.3	0.5	0.1	
17:0		0.6	0.6	0.3	
18:0	1.1	0.7	0.6	1.1	0.8
18:1 w5c	0.2	0.2	0.2	0.2	
18:1ω7c 11-methyl	8.0	10.9	11.6	8.7	6.7
18:3 ω6c (6,9,12)	0.2	0.1		0.1	
19:0 iso		0.2			
19:0 cyclo ω8c			0.8	1.8	
20:0		0.5			
20:0 iso			1.3	3.2	
20:1 ω7c		0.8		0.2	
Summed feature 3†	1.9	2.6	2.5	0.4	1.1
Summed feature 7†		0.2		0.1	
Summed feature 8†	70.4	65.7	65.8	67.4	74.8

* Summed Features are fatty acids that cannot be resolved reliably from another fatty acid using the chromatographic conditions chosen. The MIDI system groups these fatty acids together as one feature with a single percentage of the total. Summed feature 2, 12:0 aldehyde/?; Summed feature 3, 16:1 ω6c/16:1 ω7c; Summed feature 5, 18:0 ante/18:2 ω6,9c; Summed feature 8, 18:1 ω6c/18:1 ω7c.

† Data from: Yu X, Cloutier S, Tambong J, Bromfield ESP. *Int J Syst Evol Microbiol* 2014; 64: 3202–3207.

Table S5 Phenotypic characteristics of **1**. *B. ontarionense* sp. nov., A19^T, **2**. *B. oligotrophicum* S58^T, **3.** *B. denitrificans* IFAM 1005^T, **4.** *'B. aeschynomenes'* 83002^T, **5.** *B. japonicum* USDA 6^T.

**Characteristic**	**1**	**2**	**3**	**4**	**5** [a]	**Characteristic**	**1**	**2**	**3**	**4**	**5** [a]
**C-source utilization (Biolog)** [b]											
Dextrin	-	-	-	-	-	Glycyl-L-Proline	-	-	-	-	-
D-Maltose	-	-	-	-	-	L-Alanine	-	-	-	-	-
D-Trehalose	-	-	-	-	-	L-Arginine	-	-	-	-	-
D-Cellobiose	-	-	-	-	-	L-Aspartic Acid	-	-	-	+	-
Gentiobiose	-	-	-	-	-	L-Glutamic Acid	-	-	-	-	-
Sucrose	-	-	-	-	-	L-Histidine	-	-	-	-	-
D-Turanose	-	-	-	-	-	L-Pyroglutamic Acid	-	-	-	+	-
Stachyose	-	-	-	-	-	L-Serine	-	-	-	-	-
D-Raffinose	-	-	-	-	-	Pectin	-	±	-	-	-
α-D-Lactose	-	-	-	-	-	D-Galacturonic Acid	-	+	+	+	+
D-Melibiose	-	-	-	-	-	L-Galactonic Acid Lactone	-	+	+	+	-
β-Methyl-DGlucoside	-	-	-	-	-	D-Gluconic Acid	±	+	±	-	-
D-Salicin	-	-	-	-	-	D-Glucuronic Acid	-	+	+	+	+
N-Acetyl-DGlucosamine	-	-	-	-	-	Glucuronamide	-	+	+	+	+
N-Acetyl-β-DMannosamine	-	-	-	-	-	Mucic Acid	-	±	±	-	+
N-Acetyl-DGalactosamine	-	-	-	-	-	Quinic Acid	+	+	±	+	±
N-Acetyl Neuraminic Acid	-	-	-	-	-	D-Saccharic Acid	-	+	±	+	+
α-D-Glucose	±	-	-	+	-	p-HydroxyPhenylacetic Acid	-	±	-	-	-
D-Mannose	-	-	-	-	-	Methyl Pyruvate	-	±	±	+	+
D-Fructose	-	-	-	±	-	D-Lactic Acid Methyl Ester	-	±	-	-	-
D-Galactose	±	±	-	±	-	L-Lactic Acid	-	±	-	-	+
3-Methyl Glucose	-	-	-	-	-	Citric Acid	-	-	-	+	-
D-Fucose	+	+	±	+	+	α-Keto-Glutaric Acid	-	-	-	±	-
L-Fucose	-	±	±	-	-	D-Malic Acid	+	+	-	+	+
L-Rhamnose	-	-	-	-	-	L-Malic Acid	±	+	-	+	+
Inosine	-	-	-	-	-	Bromo-Succinic Acid	-	-	-	±	-
D-Sorbitol	±	+	±	±	-	Tween 40	+	+	±	±	-
D-Mannitol	±	+	+	±	-	γ-Amino-Butryric Acid	-	±	-	-	-
D-Arabitol	±	+	+	+	-	α-HydroxyButyric Acid	-	±	-	-	-
myo-Inositol	-	±	±	-	-	β-Hydroxy-D,LButyric Acid	±	+	±	+	+
Glycerol	±	+	+	+	+	α-Keto-Butyric Acid	-	-	-	-	-
D-Glucose- 6-PO4	-	±	±	-	-	Acetoacetic Acid	±	+	+	-	-
D-Fructose- 6-PO4	-	±	+	-	-	Propionic Acid	+	+	+	-	-
D-Aspartic Acid	-	±	-	-	-	Acetic Acid	+	+	+	-	-
Gelatin	-	±	-	-	-	Formic Acid	+	+	v	+	+
**Chemical Sensitivity (Biolog)** [b]											
1% Sodium Lactate	±	±	-	-	+	Vancomycin	-	±	±	+	+
Fusidic Acid	-	-	-	-	-	Tetrazolium Violet	±	+	+	+	+
D-Serine	-	-	-	-	-	Tetrazolium Blue	±	+	+	+	+
Troleandomycin	±	+	-	-	-	Nalidixic Acid	+	±	-	±	+
Rifamycin SV	±	+	±	+	+	Lithium Chloride	-	-	-	-	-
Minocycline	±	+	±	+	+	Potassium Tellurite	-	-	-	-	-
Lincomycin	+	-	+	+	+	Aztreonam	+	±	+	+	+
Guanidine HCl	-	-	-	-	-	Sodium Butyrate	-	+	-	-	-
Niaproof 4	-	-	-	-	-	Sodium Bromate	-	-	-	-	-

[a] Data from Bromfield ESP, Cloutier S and Nguyen HDT. *Int J Syst Evol Microbiol* 2020; 70: 442-449.

[b] BIOLOG GEN III MicroPlates (120 hours incubation at 28 ºC): +, Positive; ±, weak; −, negative; v, variable. ND, not determined. Values are based on three replicates.

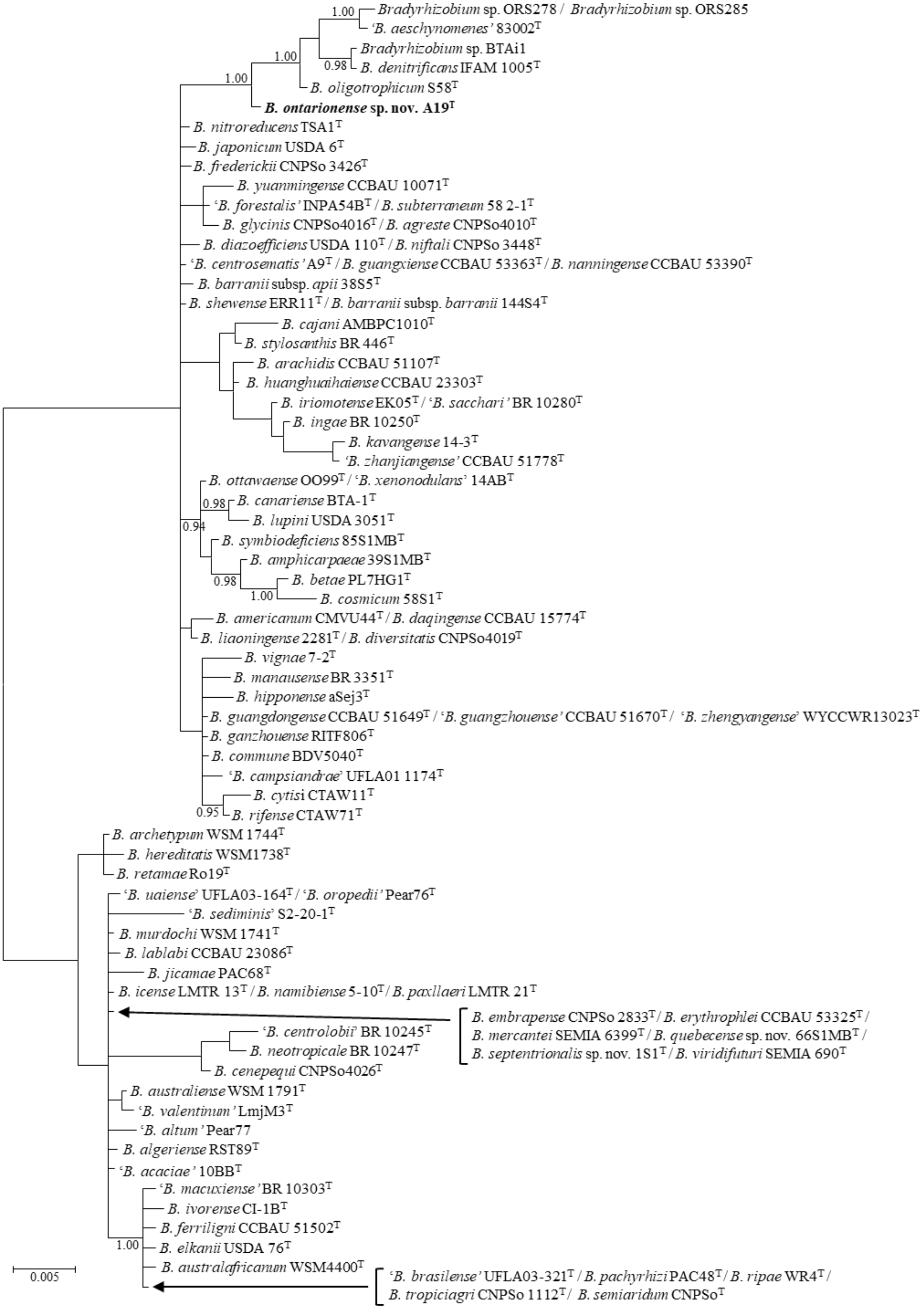

1.00
Bradyrhizobium sp. ORS278 / *Bradyrhizobium* sp. ORS285
'*B. aeschynomenes*' 83002^{T}
1.00
Bradyrhizobium sp. BTAi1
0.98
B. denitrificans IFAM 1005^{T}
1.00
B. oligotrophicum S58^{T}
B. ontarionense sp. nov. A19^{T}
B. nitroreducens TSA1^{T}
B. japonicum USDA 6^{T}
B. frederickii CNPSo 3426^{T}
B. yuanmingense CCBAU 10071^{T}
'*B. forestalis*' INPA54B^{T} / *B. subterraneum* 58 2-1^{T}
B. glycinis CNPSo4016^{T} / *B. agreste* CNPSo4010^{T}
B. diazoefficiens USDA 110^{T} / *B. niftali* CNPSo 3448^{T}
'*B. centrosematis*' A9^{T} / *B. guangxiense* CCBAU 53363^{T} / *B. nanningense* CCBAU 53390^{T}
B. barranii subsp. *apii* 38S5^{T}
B. shewense ERR11^{T} / *B. barranii* subsp. *barranii* 144S4^{T}
B. cajani AMBPC1010^{T}
B. stylosanthis BR 446^{T}
B. arachidis CCBAU 51107^{T}
B. huanghuaihaiense CCBAU 23303^{T}
B. iriomotense EK05^{T} / '*B. sacchari*' BR 10280^{T}
B. ingae BR 10250^{T}
B. kavangense 14-3^{T}
'*B. zhanjiangense*' CCBAU 51778^{T}
B. ottawaense OO99^{T} / '*B. xenonodulans*' 14AB^{T}
0.98
B. canariense BTA-1^{T}
0.94
B. lupini USDA 3051^{T}
B. symbiodeficiens 85S1MB^{T}
B. amphicarpaeae 39S1MB^{T}
0.98
B. betae PL7HG1^{T}
1.00
B. cosmicum 58S1^{T}
B. americanum CMVU44^{T} / *B. daqingense* CCBAU 15774^{T}
B. liaoningense 2281^{T} / *B. diversitatis* CNPSo4019^{T}
B. vignae 7-2^{T}
B. manausense BR 3351^{T}
B. hipponense aSej3^{T}
B. guangdongense CCBAU 51649^{T} / '*B. guangzhouense*' CCBAU 51670^{T} / '*B. zhengyangense*' WYCCWR13023^{T}
B. ganzhouense RITF806^{T}
B. commune BDV5040^{T}
'*B. campsiandrae*' UFLA01 1174^{T}
B. cytisi CTAW11^{T}
0.95
B. rifense CTAW71^{T}
B. archetypum WSM 1744^{T}
B. hereditatis WSM1738^{T}
B. retamae Ro19^{T}
'*B. uaiense*' UFLA03-164^{T} / '*B. oropedii*' Pear76^{T}
'*B. sediminis*' S2-20-1^{T}
B. murdochi WSM 1741^{T}
B. lablabi CCBAU 23086^{T}
B. jicamae PAC68^{T}
B. icense LMTR 13^{T} / *B. namibiense* 5-10^{T} / *B. paxllaeri* LMTR 21^{T}
B. embrapense CNPSo 2833^{T} / *B. erythrophlei* CCBAU 53325^{T} /
B. mercantei SEMIA 6399^{T} / *B. quebecense* sp. nov. 66S1MB^{T} /
B. septentrionalis sp. nov. 1S1^{T} / *B. viridifuturi* SEMIA 690^{T}
'*B. centrolobii*' BR 10245^{T}
B. neotropicale BR 10247^{T}
B. cenepequi CNPSo4026^{T}
B. australiense WSM 1791^{T}
'*B. valentinum*' LmjM3^{T}
'*B. altum*' Pear77
B. algeriense RST89^{T}
'*B. acaciae*' 10BB^{T}
'*B. macuxiense*' BR 10303^{T}
B. ivorense CI-1B^{T}
B. ferriligni CCBAU 51502^{T}
1.00
B. elkanii USDA 76^{T}
B. australafricanum WSM4400^{T}
'*B. brasilense*' UFLA03-321^{T} / *B. pachyrhizi* PAC48^{T} / *B. ripae* WR4^{T} /
B. tropiciagri CNPSo 1112^{T} / *B. semiaridum* CNPSo^{T}
0.005

Figure S1. Bayesian tree of 16S rRNA gene sequences of *Bradyrhizobium ontarionense* sp. nov. strain A19^T and reference strains. Best fit substitution model: HKY + G + I. Posterior probabilities ≥ 0.9 are shown. Scale, expected number of substitutions per site. To include all species of *Bradyrhizobium* in the tree, alignment lengths of sequences were trimmed to 1300 bp.

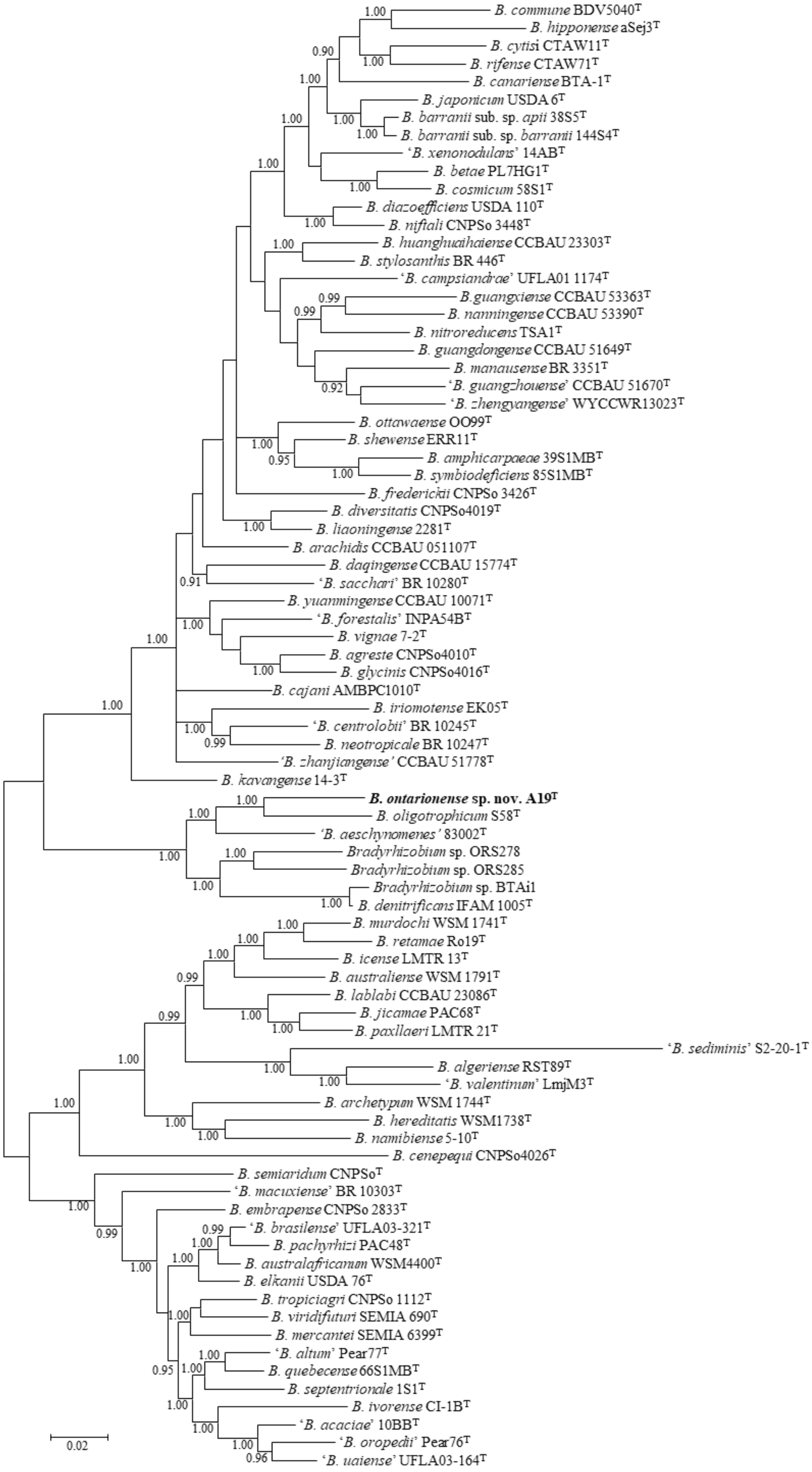

B. commune BDV5040^{T}
B. hipponense aSej3^{T}
B. cytisi CTAW11^{T}
B. rifense CTAW71^{T}
B. canariense BTA-1^{T}
B. japonicum USDA 6^{T}
B. barranii sub. sp. apii 38S5^{T}
B. barranii sub. sp. barranii 144S4^{T}
'B. xenonodulans' 14AB^{T}
B. betae PL7HG1^{T}
B. cosmicum 58S1^{T}
B. diazoefficiens USDA 110^{T}
B. niftali CNPSo 3448^{T}
B. huanghuaihaiense CCBAU 23303^{T}
B. stylosanthis BR 446^{T}
'B. campsiandrae' UFLA01 1174^{T}
B. guangxiense CCBAU 53363^{T}
B. nanningense CCBAU 53390^{T}
B. nitroreducens TSA1^{T}
B. guangdongense CCBAU 51649^{T}
B. manausense BR 3351^{T}
'B. guangzhouense' CCBAU 51670^{T}
'B. zhengyangense' WYCCWR13023^{T}
B. ottawaense OO99^{T}
B. shewense ERR11^{T}
B. amphicarpaeae 39S1MB^{T}
B. symbiodeficiens 85S1MB^{T}
B. frederickii CNPSo 3426^{T}
B. diversitatis CNPSo4019^{T}
B. liaoningense 2281^{T}
B. arachidis CCBAU 051107^{T}
B. daqingense CCBAU 15774^{T}
'B. sacchari' BR 10280^{T}
B. yuanmingense CCBAU 10071^{T}
'B. forestalis' INPA54B^{T}
B. vignae 7-2^{T}
B. agreste CNPSo4010^{T}
B. glycinis CNPSo4016^{T}
B. cajani AMBPC1010^{T}
B. iriomotense EK05^{T}
'B. centrolobii' BR 10245^{T}
B. neotropicale BR 10247^{T}
'B. zhanjiangense' CCBAU 51778^{T}
B. kavangense 14-3^{T}
B. ontarionense sp. nov. A19^{T}
B. oligotrophicum S58^{T}
'B. aeschynomenes' 83002^{T}
Bradyrhizobium sp. ORS278
Bradyrhizobium sp. ORS285
Bradyrhizobium sp. BTAi1
B. denitrificans IFAM 1005^{T}
B. murdochi WSM 1741^{T}
B. retamae Ro19^{T}
B. icense LMTR 13^{T}
B. australiense WSM 1791^{T}
B. lablabi CCBAU 23086^{T}
B. jicamae PAC68^{T}
B. paxllaeri LMTR 21^{T}
'B. sediminis' S2-20-1^{T}
B. algeriense RST89^{T}
'B. valentinum' LmjM3^{T}
B. archetypum WSM 1744^{T}
B. hereditatis WSM1738^{T}
B. namibiense 5-10^{T}
B. cenepequi CNPSo4026^{T}
B. semiaridum CNPSo^{T}
'B. macuxiense' BR 10303^{T}
B. embrapense CNPSo 2833^{T}
'B. brasilense' UFLA03-321^{T}
B. pachyrhizi PAC48^{T}
B. australafricanum WSM4400^{T}
B. elkanii USDA 76^{T}
B. tropiciagri CNPSo 1112^{T}
B. viridifuturi SEMIA 690^{T}
B. mercantei SEMIA 6399^{T}
'B. altum' Pear77^{T}
B. quebecense 66S1MB^{T}
B. septentrionale 1S1^{T}
B. ivorense CI-1B^{T}
'B. acaciae' 10BB^{T}
'B. oropedii' Pear76^{T}
'B. uaiense' UFLA03-164^{T}
0.02

Figure S2 Bayesian phylogenetic tree (GTR+G+I substitution model) of *atpD–glnII–recA–gyrB–rpoB* concatenated partial housekeeping gene sequences (2679 bp alignment length) for *Bradyrhizobium ontarionense* sp. nov. strain A19^T and reference taxa of the genus *Bradyrhizobium*. Posterior probabilities ≥ 0.9 are shown. Bar, expected substitutions per site.

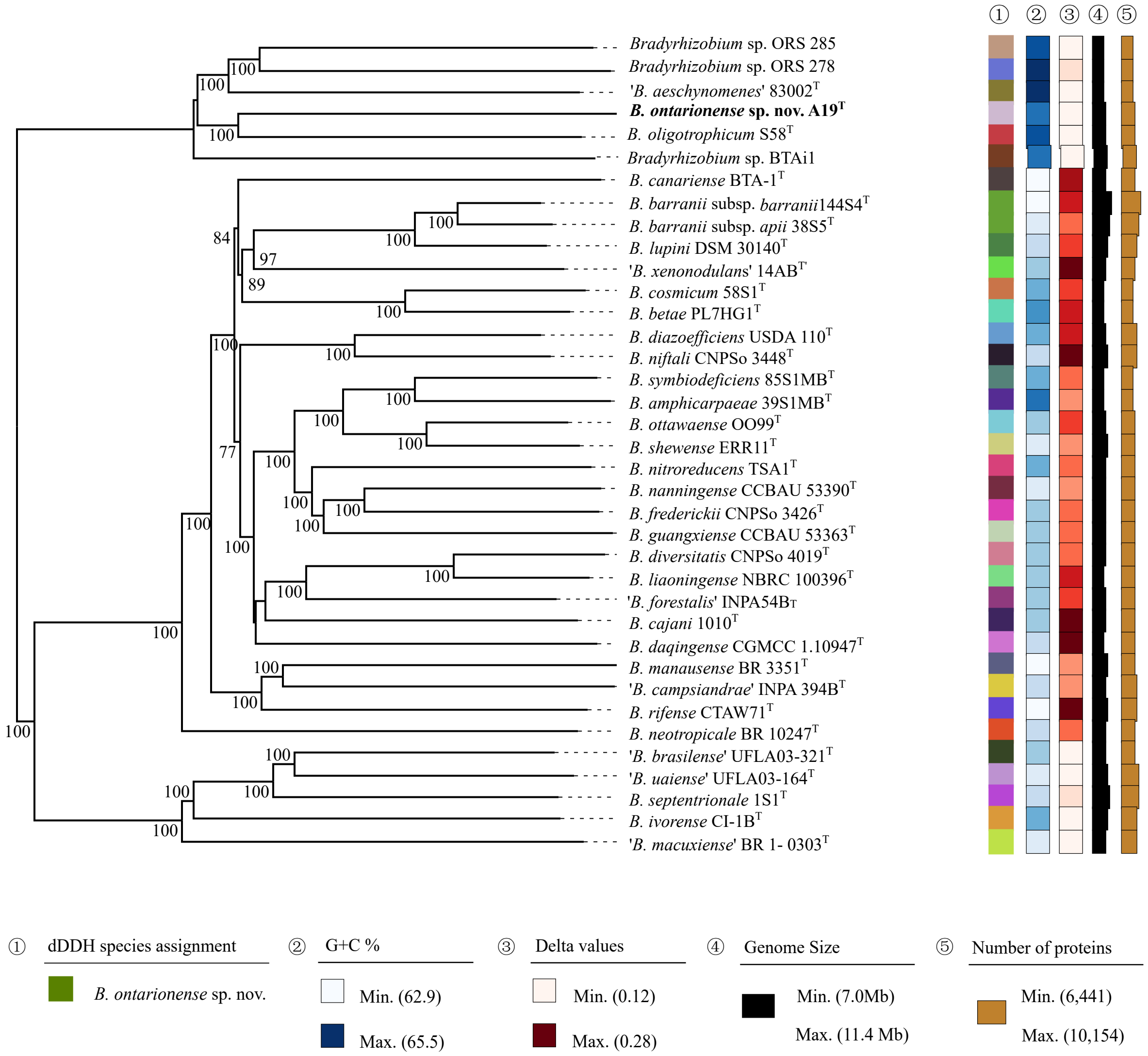
① ② ③ ④ ⑤
Bradyrhizobium sp. ORS 285
Bradyrhizobium sp. ORS 278
'B. aeschynomenes' 83002T
B. ontarionense sp. nov. A19T
B. oligotrophicum S58T
Bradyrhizobium sp. BTAi1
B. canariense BTA-1T
B. barranii subsp. barranii144S4T
B. barranii subsp. apii 38S5T
B. lupini DSM 30140T
'B. xenonodulans' 14ABT
B. cosmicum 58S1T
B. betae PL7HG1T
B. diazoefficiens USDA 110T
B. niftali CNPSo 3448T
B. symbiodeficiens 85S1MBT
B. amphicarpaeae 39S1MBT
B. ottawaense OO99T
B. shewense ERR11T
B. nitroreducens TSA1T
B. nanningense CCBAU 53390T
B. frederickii CNPSo 3426T
B. guangxiense CCBAU 53363T
B. diversitatis CNPSo 4019T
B. liaoningense NBRC 100396T
'B. forestalis' INPA54BT
B. cajani 1010T
B. daqingense CGMCC 1.10947T
B. manausense BR 3351T
'B. campsiandrae' INPA 394BT
B. rifense CTAW71T
B. neotropicale BR 10247T
'B. brasilense' UFLA03-321T
'B. uaiense' UFLA03-164T
B. septentrionale 1S1T
B. ivorense CI-1BT
'B. macuxiense' BR 1- 0303T
① dDDH species assignment
B. ontarionense sp. nov.
② G+C %
Min. (62.9)
Max. (65.5)
③ Delta values
Min. (0.12)
Max. (0.28)
④ Genome Size
Min. (7.0Mb)
Max. (11.4 Mb)
⑤ Number of proteins
Min. (6,441)
Max. (10,154)

Figure S3 Phylogenomic tree based on TYGS implementation showing *Bradyrhizobium ontarionense* sp. nov. A19^{T} and most closely related reference taxa of the genus *Bradyrhizobium*. The tree was inferred with FastME 2.1.6.1 (Ciufo et al. 2018) from Genome Blast Distance Phylogeny (GBDP) distances calculated from genome sequences. Branch lengths are scaled according to GBDP distance formula d5. The numbers above branches represent GBDP pseudo-bootstrap support values (≥ 70%) from 100 replications, with an average branch support of 94%. The tree is rooted at the mid-point. Leaf labels are annotated by affiliation to species (1), genomic G+C content (2), delta values (3), overall genome sequence length (4) and number of proteins (5). Delta statistics permit assessment of accuracy in terms of tree-likeness; the lower the delta value, the greater the accuracy (Holland et al. 2002).

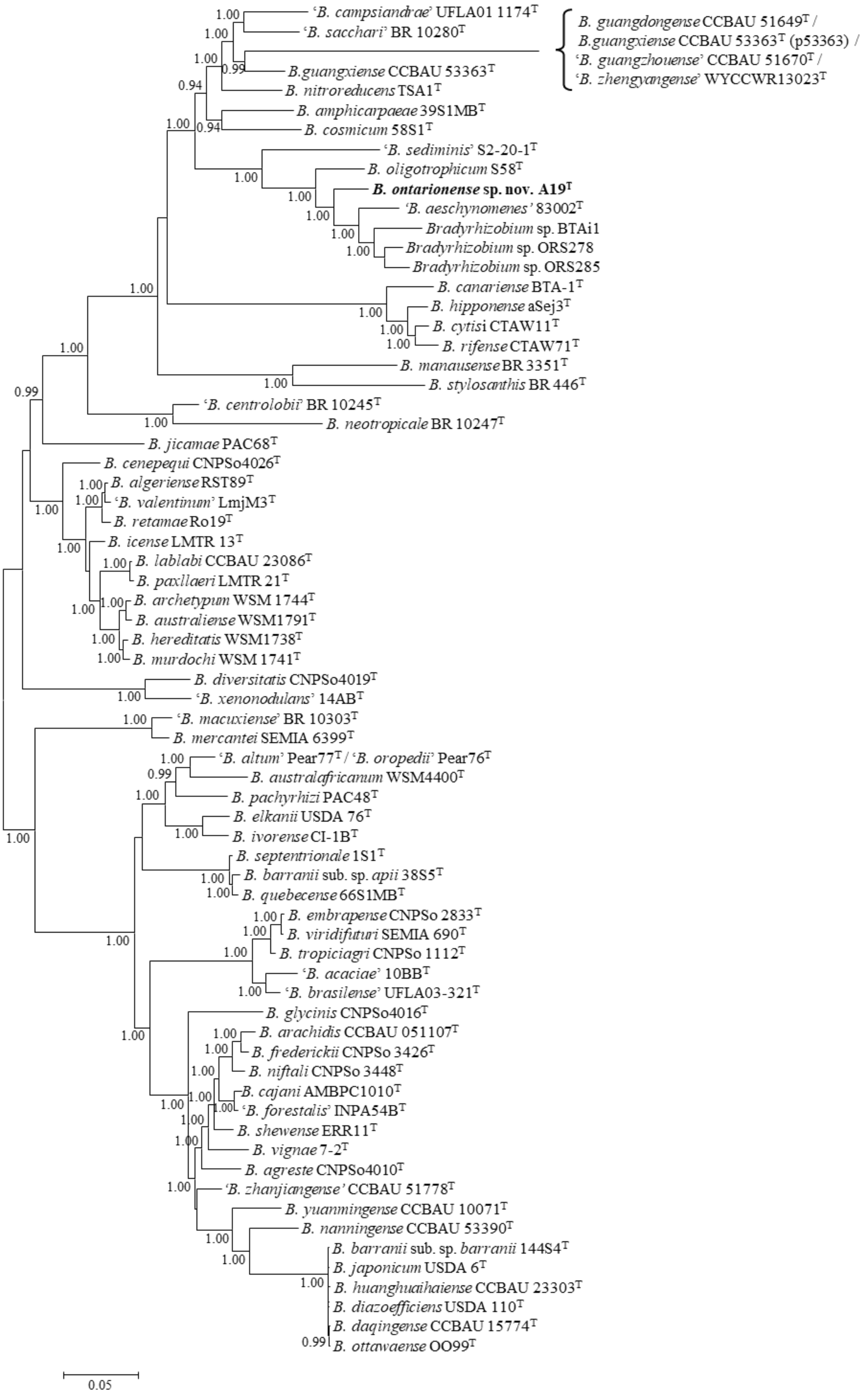
'B. campsiandrae' UFLA01 1174T
'B. sacchari' BR 10280T
B. guangdongense CCBAU 51649T /
B.guangxiense CCBAU 53363T (p53363) /
'B. guangzhouense' CCBAU 51670T /
'B. zhengyangense' WYCCWR13023T
B.guangxiense CCBAU 53363T
B. nitroreducens TSA1T
B. amphicarpaeae 39S1MBT
B. cosmicum 58S1T
'B. sediminis' S2-20-1T
B. oligotrophicum S58T
B. ontarionense sp. nov. A19T
'B. aeschynomenes' 83002T
Bradyrhizobium sp. BTAi1
Bradyrhizobium sp. ORS278
Bradyrhizobium sp. ORS285
B. canariense BTA-1T
B. hipponense aSej3T
B. cytisi CTAW11T
B. rifense CTAW71T
B. manausense BR 3351T
B. stylosanthis BR 446T
'B. centrolobii' BR 10245T
B. neotropicale BR 10247T
B. jicamae PAC68T
B. cenepequi CNPSo4026T
B. algeriense RST89T
'B. valentinum' LmjM3T
B. retamae Ro19T
B. icense LMTR 13T
B. lablabi CCBAU 23086T
B. paxllaeri LMTR 21T
B. archetypum WSM 1744T
B. australiense WSM1791T
B. hereditatis WSM1738T
B. murdochi WSM 1741T
B. diversitatis CNPSo4019T
'B. xenonodulans' 14ABT
'B. macuxiense' BR 10303T
B. mercantei SEMIA 6399T
'B. altum' Pear77T / 'B. oropedii' Pear76T
B. australafricanum WSM4400T
B. pachyrhizi PAC48T
B. elkanii USDA 76T
B. ivorense CI-1BT
B. septentrionale 1S1T
B. barranii sub. sp. apii 38S5T
B. quebecense 66S1MBT
B. embrapense CNPSo 2833T
B. viridifuturi SEMIA 690T
B. tropiciagri CNPSo 1112T
'B. acaciae' 10BBT
'B. brasilense' UFLA03-321T
B. glycinis CNPSo4016T
B. arachidis CCBAU 051107T
B. frederickii CNPSo 3426T
B. niftali CNPSo 3448T
B. cajani AMBPC1010T
'B. forestalis' INPA54BT
B. shewense ERR11T
B. vignae 7-2T
B. agreste CNPSo4010T
'B. zhanjiangense' CCBAU 51778T
B. yuanmingense CCBAU 10071T
B. nanningense CCBAU 53390T
B. barranii sub. sp. barranii 144S4T
B. japonicum USDA 6T
B. huanghuaihaiense CCBAU 23303T
B. diazoefficiens USDA 110T
B. daqingense CCBAU 15774T
B. ottawaense OO99T
0.05

Figure S4. Bayesian tree of concatenated *nifHDK* gene sequences (3479bp) of *Bradyrhizobium ontarionense* sp. nov. strain A19^T and reference taxa of the genus *Bradyrhizobium*. Best fit substitution model, GTR+G+I. Posterior probabilities ≥ 0.9 are shown. Bar, expected substitutions per site.

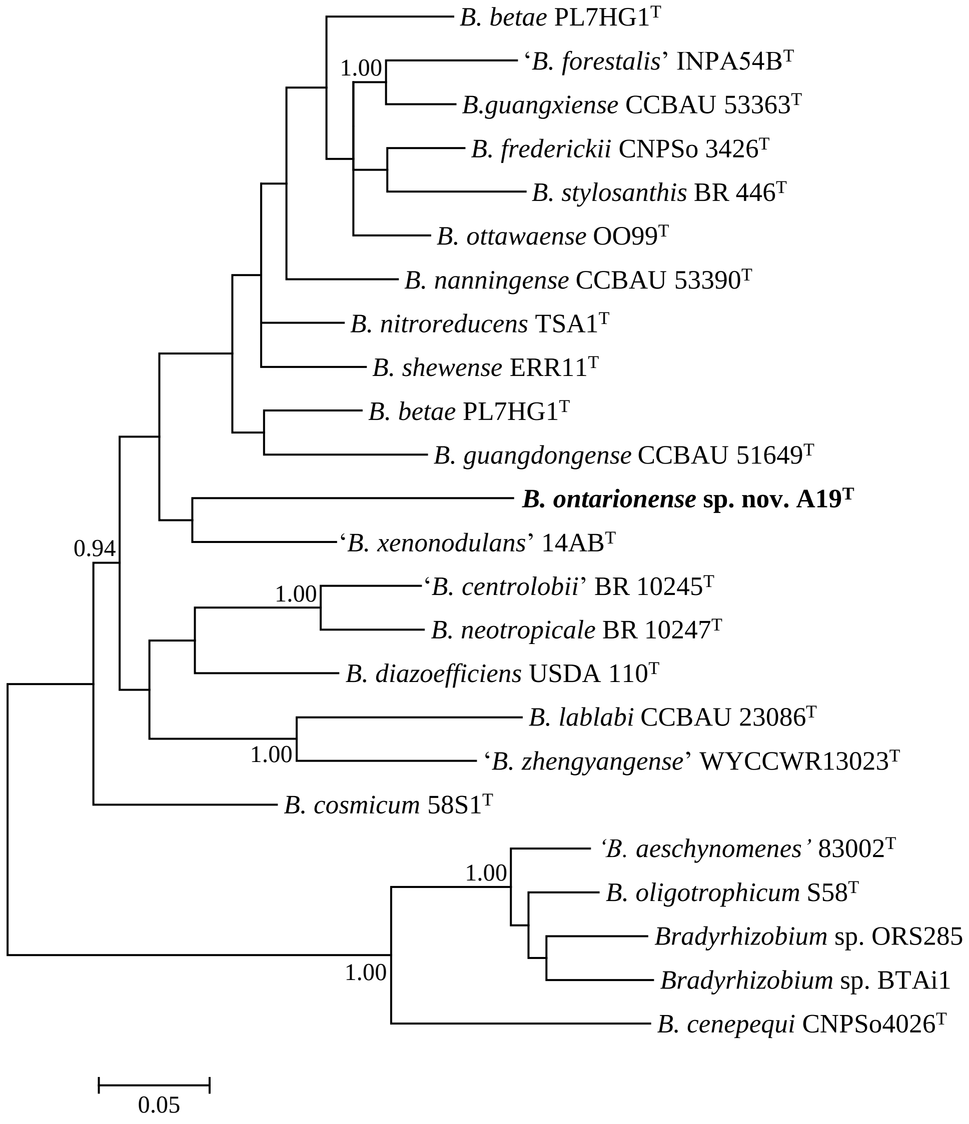

Figure S5 Bayesian phylogenetic tree (GTR+G+I substitution model) of nitrous oxide reductase (*nosZ*) gene sequences (1748 bp) for *Bradyrhizobium ontarionense* sp. nov. strain A19^T and reference taxa consisting of type strains of described species of the genus *Bradyrhizobium*. Posterior probabilities ≥ 0.90 are indicated. Bar represents expected substitutions per site.

(A)

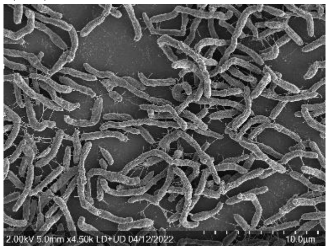

(B)

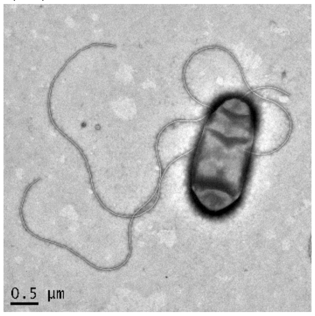

(C)

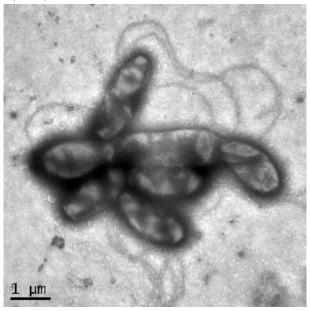

Figure S6 Scanning (A) and transmission (B and C) electron microscope images showing morphological features of cells of *Bradyrhizobium ontarionense* sp. nov A19^{T}. Scale bars (µm) are indicated in the images.
